# Incidence of and Risk Factors for Paradoxical Psoriasis or Psoriasiform Lesions in Inflammatory Bowel Disease Patients Receiving Anti-TNF Therapy: Systematic Review With Meta-Analysis

**DOI:** 10.3389/fimmu.2022.847160

**Published:** 2022-03-01

**Authors:** Wenhui Xie, Shiyu Xiao, Hong Huang, Zhuoli Zhang

**Affiliations:** ^1^ Department of Rheumatology and Clinical Immunology, Peking University First Hospital, Beijing, China; ^2^ Department of Gastroenterology, Peking University Third Hospital, Beijing, China

**Keywords:** inflammatory bowel disease, anti-tumor necrosis factor, psoriasis, psoriasiform lesions, risk factors, meta-analysis, incidence

## Abstract

**Background:**

Paradoxical psoriasis or psoriasiform lesions induced by anti-tumor necrosis factor (anti-TNF) therapies receive increasing attention worldwide. However, no comprehensive meta-analysis investigating the incidence estimates and risk factors for anti-TNF-induced psoriasis is currently available. We aimed to precisely quantify its incidence as well as risk factors in patients with inflammatory bowel disease (IBD).

**Methods:**

This study was registered on PROSPERO database under review registration number CRD42021233695. The electronic databases PubMed, EMBASE, and the Cochrane library were comprehensively searched for observational studies published as full-length papers in English and reporting the incidence and/or predictors for psoriasis or psoriasiform lesions in IBD patients. A random-effects meta-analysis was performed to calculate the pooled incidence. Pooled odds ratio (OR) and 95% confidence interval for potential predictors were combined using a fixed-effects or random-effects model.

**Results:**

In total, 30 articles comprising 24,547 IBD patients treated by anti-TNF were finally included. The overall pooled incidence of psoriasis and/or psoriasiform lesions following anti-TNF therapy was 6.0% (5.0–7.0%; *I* ^2^ = 93.9%), with 6.9% (5.1–8.7%; *I* ^2^ = 92.4%) for psoriasiform lesions and 4.6% (3.6–5.6%; *I* ^2^ = 93.9%) for psoriasis. Multivariable meta-regression analysis indicated regions and populations that significantly contributed to the heterogeneity. A statistically higher risk for psoriasis or psoriasiform lesions during anti-TNF therapy was observed in female patients (OR 1.46, 1.23–1.73), those who are at a younger age at anti-TNF initiation (OR 1.03, 1.00–1.05), smokers (OR 1.97, 1.56–2.48), ileocolonic Crohn’s disease patients (OR 1.48, 1.03–2.13), and those who are using adalimumab or certolizumab (*vs*. infliximab) (OR: 1.48 and 2.87 respectively).

**Conclusions:**

The incidence of psoriasis or psoriasiform lesions was not uncommon in IBD patients following anti-TNF therapy. Female, younger age, smoker, ileocolonic Crohn’s disease, and the types of anti-TNF were significantly associated with such risk. These findings may help gastroenterologists to make more individualized decisions and understand the mechanisms of this paradoxical phenomenon.

**Systematic Review Registration:**

https://www.crd.york.ac.uk/PROSPERO/display_record.php?RecordID=233695, identifier CRD42021233695.

**Graphical Abstract:**
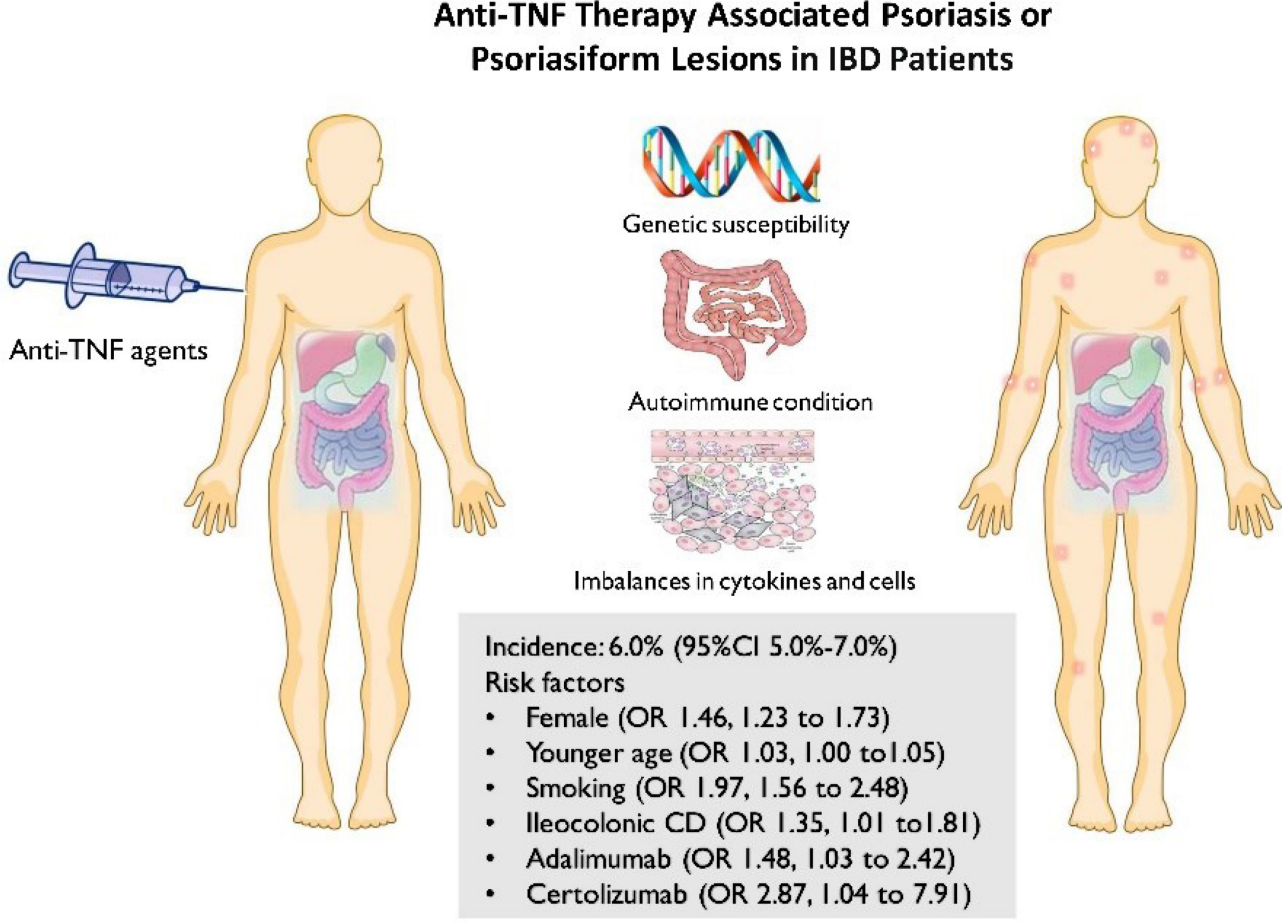


## Introduction

Inflammatory bowel diseases (IBDs) are chronic inflammatory disorders of the gastrointestinal tract that affect approximately 10 million patients worldwide (1, 2). The introduction of biologics has dramatically transformed the therapeutic landscape of IBD. Among these, anti-tumor necrosis factor (anti-TNF), such as infliximab and adalimumab, are most extensively used in daily practice for a couple of decades (3). Accumulative evidence has demonstrated that anti-TNF can control the disease activity rapidly, exert a steroid-sparing effect, promote mucosal healing, improve the quality of life, and reduce the risk of surgery as well (3, 4).

With increasing use of these agents, however, some paradoxical inflammations, involving the skin, joints and lungs, have been described and received increasing attention in recent years (5). Of these, paradoxical psoriasis or psoriasiform lesion induced by anti-TNF therapies is one of the most extended concerned topics worldwide. Generally, anti-TNF treatments are commonly used for psoriasis therapy, but psoriasis and psoriasiform skin lesions are sometimes observed in IBD patients receiving anti-TNF therapies. Overall, IBD patients treated with anti-TNF therapy have a 2.4-fold increased risk of paradoxical psoriasis compared with nonusers of anti-TNF (6). Meanwhile, there is high inconsistency in the results of previous studies on the incidence of psoriasis or psoriasiform lesions in IBD patients with exposure to anti-TNF, varying from 1% (6) to more than 30% (7). The relatively small sample sizes and limited number of events lead to significant variation and imprecise incidence estimates and further preclude robust conclusions to be drawn from any of the individual studies. On the other hand, currently, the knowledge about the risk factor for psoriasis or psoriasiform lesions secondary to anti-TNF therapy in IBD patients is limited and contradictory—for example, some studies have shown gender, smoking, and concomitant immunosuppressive agents to be associated with an increased risk of developing anti-TNF-induced psoriasis, while others have not (8–16).

To date, no comprehensive meta-analysis investigating the incidence estimates and risk factors for anti-TNF-induced psoriasis is currently available. To fill the gap, the present study is intended to precisely quantify the incidence of and risk factors for developing anti-TNF-induced psoriasis or psoriasiform lesions in IBD patients.

## Materials and Methods

This article was carried out and reported in accordance with the Preferred Reporting Items for Systematic Reviews and Meta-analysis (17). The methods were stipulated in a protocol that was registered with PROSPERO (CRD42021233695).

### Literature Search and Inclusion Criteria

A literature search of English language publications was performed using the electronic databases PubMed, EMBASE, and the Cochrane Library from database inception to February 9, 2021. The search strategy was designed and conducted by an experienced medical librarian with input from the study investigators. The studies were identified by combining three search themes: the first theme, inflammatory bowel disease; the second theme, anti-TNF; and the third theme, a combination of the following terms: psoriasis, psoriasiform, dermatological, skin, and cutaneous. The detailed search strategies are available in **Supplementary Appendix S1**.

Studies were included if they were on IBD patients of all ages (including children) receiving anti-TNF treatment, were cohort studies or case–control studies, reported the incidence of and/or risk factors for psoriasis or psoriasiform lesions in IBD patients, and were full-text English articles. When duplicate publications were identified, only the article with the newest and most comprehensive information was included. We excluded studies with insufficient data of interest (such as those only presenting all dermatological events), meeting abstract, case report, editorial, review, or nonhuman investigations. Two investigators (WX and SX) independently evaluated the eligibility, and any discrepancy throughout was resolved by a third investigator (ZZ).

### Data Extraction and Outcome Assessment

Data extraction of the eligible studies was conducted by two independent review authors (WX and SX) using piloted data extraction sheets: first author, publication year, country/countries, study design, data sources, setting, study period, the diagnosis of IBD, psoriasis, sample size, time period of observations, patients’ demographics and clinical characteristics, number of patients developing psoriasis or psoriasiform lesions, risk factor of interests, and risk estimates. The methodological quality of each study was rated by the Newcastle–Ottawa Scale (NOS) which consists of three factors: patient selection (0–4 points), comparability of the study groups (0–2 points), and assessment of outcome (0–3 points) (18). All relevant studies were scored from 0 to 9 on the NOS to determine the study quality.

### Data Synthesis and Analysis

All calculations and graphs were performed using Stata Statistical Software version 13.0. The incidence of psoriasis or psoriasiform lesions in IBD patients treated with anti-TNF therapy was pooled. The levels of heterogeneity were assessed by the *I* ^2^ statistic (*I*² >50% was considered as a statistically significant heterogeneity). If severe heterogeneity was present at *I* ^2^ >50%, the random-effects model (DerSimonian and Laird method) was chosen; otherwise, the fixed-effects model was adopted (Mantel–Haenszel method). The sources of heterogeneity were explored by using subgroup and meta-regression analyses. On the other hand, to explore the risk factors for developing psoriasis or psoriasiform lesions, the patients’ demographics and clinical characteristics (sex, age, smoking, disease phenotype, type of anti-TNF therapy, *etc.*) were compared between IBD patients with and without psoriasis/psoriasiform lesions during anti-TNF therapy, if possible. Pooled odds ratios (ORs) with 95% CI were calculated as an effect measure. We extracted the risk estimates that were adjusted for most variables. When no raw data were available, relative risks and hazard ratios were taken as good estimates of OR, in line with previous reports (19–21). Sensitivity analyses were performed to assess the robustness of estimates. Graphical symmetry with funnel plot, as well as with Begg’s and Egger’s statistical tests, was produced to help detect publication bias. Trim-and-filled method was performed in the case of potential publication bias. A two-sided *P*-value <0.05 was considered statistically significant.

## Results

### Study Selection and Characteristics

The study selection process is shown in **Supplementary Figure S1**. Initially, we retrieved 11,467 citations, of which 107 full-text articles were eligible for inclusion (6–16, 22–40). The characteristics of the included studies are presented in **Table 1**. In total, 30 citations were published between 2009 and 2020. All papers were based on retrospective or prospective cohorts, and majority of them originated from Europe and the USA. Psoriasis or psoriasiform lesions diagnosis was mostly judged by the treating gastroenterologist and/or dermatologist (biopsy if necessary) (8, 10, 13–16, 22, 26, 29–31, 33–37). The median NOS score in the included studies was 6, ranging from 5 to 8 (**Supplementary Table S1**).

**Table 1:** Characteristics of the studies included in the meta-analysis.

Author, Reference	Year	Country	Data source	Study design	Enrollment period	Population	Number of IBD	Outcome	Diagnosis method	Number of events
Fidder et al. (22)	2009	Belgium	Monocenter	Retrospective	1994–2008	Adult	743	Psoriasiform lesions	Patient or treating physician	39
Rahier et al. (23)	2010	France	Multicenter	Retrospective	2004–2009	Adult	562	Psoriasiform lesions	NA	62
Baumgart et al. (24)	2011	Germany	Monocenter	Prospective	NA	Mixed	50	Psoriasiform lesions	NA	6
Hiremath et al. (25)	2011	USA	Monocenter	Retrospective	NA	Children	73	Psoriasis	NA	6
Guerra et al. (26)	2012	Spain	Multicenter	Retrospective	NA	Mixed	1,294	Psoriasis	Gastroenterologists and dermatologists; biopsy if necessary	21
Salgueiro et al. (27)	2013	Portugal	Monocenter	Retrospective	2002–2012	NA	132	Psoriasis	NA	11
Sherlock et al. (28)	2013	Canada	Monocenter	Retrospective	2000–2010	Children	172	Psoriasiform lesions	NA	18
Afzali et al. (29)	2014	USA	Monocenter	Retrospective	1998–2011	Mixed	1,004	Psoriasiform lesions	Gastroenterologists	27
Mälkönen et al. (7)	2014	Finland	Monocenter	Prospective	2011–2013	Children	84	Psoriasiform lesions	NA	25
Tillack et al. (30)	2014	Germany	Monocenter	Prospective	2010–2011	Adult	434	Psoriasiform lesions	Dermatologists	21
Włodarczyk et al. (31)	2014	Poland	Monocenter	Prospective	2012–2013	Adult	30	Psoriasiform lesions	Gastroenterologists and dermatologists	8
Pugliese et al. (32)	2015	Italy	Monocenter	Retrospective	2008–2013	Adult	402	Psoriasis		42
Fréling et al. (33)	2015	France	Monocenter	Retrospective	2000–2011	Adult	583	Psoriasiform lesions	Dermatologists	59
George et al. (34)	2015	USA	Monocenter	Retrospective	2004–2013	Mixed	72	Psoriasiform lesions	Dermatologists	18
Huang et al. (35)	2015	Canada	Monocenter	Retrospective	2013	Adult	71	Psoriasis	Gastroenterologists and dermatologist	2
Soh et al. (36)	2015	Republic of Korea	Monocenter	Retrospective	2002–2013	NA	500	Psoriasiform lesions	Dermatologist	13
Cleynen et al. (37)	2016	Belgium	Monocenter	Retrospective	1994–2009	Adult	917	Psoriasiform lesions, psoriasis	Dermatologist’s clinical diagnosis (biopsy 42%)	81
Guerra et al. (38)	2016	Spain	Multicenter	Retrospective	Inception–2015	Mixed	7,415	Psoriasis	NA	125
Hellström et al. (39)	2016	Finland	Monocenter	Prospective	2013–2014	Adult	118	Psoriasiform lesions	NA	7
Protic et al. (40)	2016	Switzerland	Monocenter	Retrospective	2010–2013	NA	269	Psoriasis	NA	23
Vedak et al. (8)	2016	USA	Monocenter	Retrospective	2005–2014	Adult	765	Psoriasis	Dermatologists	35
Jeyarajah et al. (9)	2017	Ireland	Monocenter	Retrospective	2000–2015	NA	403	Psoriasis	NA	8
Peer et al. (10)	2017	Australia	Monocenter	Retrospective	2009–2013	Adult	270	Psoriasiform lesions	Dermatologist and biopsy	10
Andrade et al. (11)	2018	Portugal	Monocenter	Retrospective	2005–2015	Mixed	732	Psoriasis	NA	39
Bae et al. (6)	2018	Korea	Nationwide	Retrospective	2007–2016	Mixed	5,428	Psoriasis	NA	62
Sridhar et al. (12)	2018	USA	Monocenter	Retrospective	2010–2015	Children	409	Psoriasis	NA	33
Weizman et al. (13)	2018	Canada	Monocenter	Retrospective	2004–2016	Mixed	676	Psoriasis	Dermatologists and biopsy	72
Courbette et al. (14)	2019	France	Monocenter	Retrospective	2002–2014	Children	147	Psoriasis	Dermatologists	20
Cossio et al. (15)	2020	Canada	Monocenter	Retrospective	2013–2016	Children	343	Psoriasis	Dermatologists	20
Ya et al. (16)	2020	USA	Monocenter	Retrospective	2003–2015	Adult	97	Psoriasis	Dermatologist and biopsy	97

NA, not available; IBD, inflammatory bowel disease.

### The Incidence of Psoriasis or Psoriasiform Lesions

**Figure 1:**
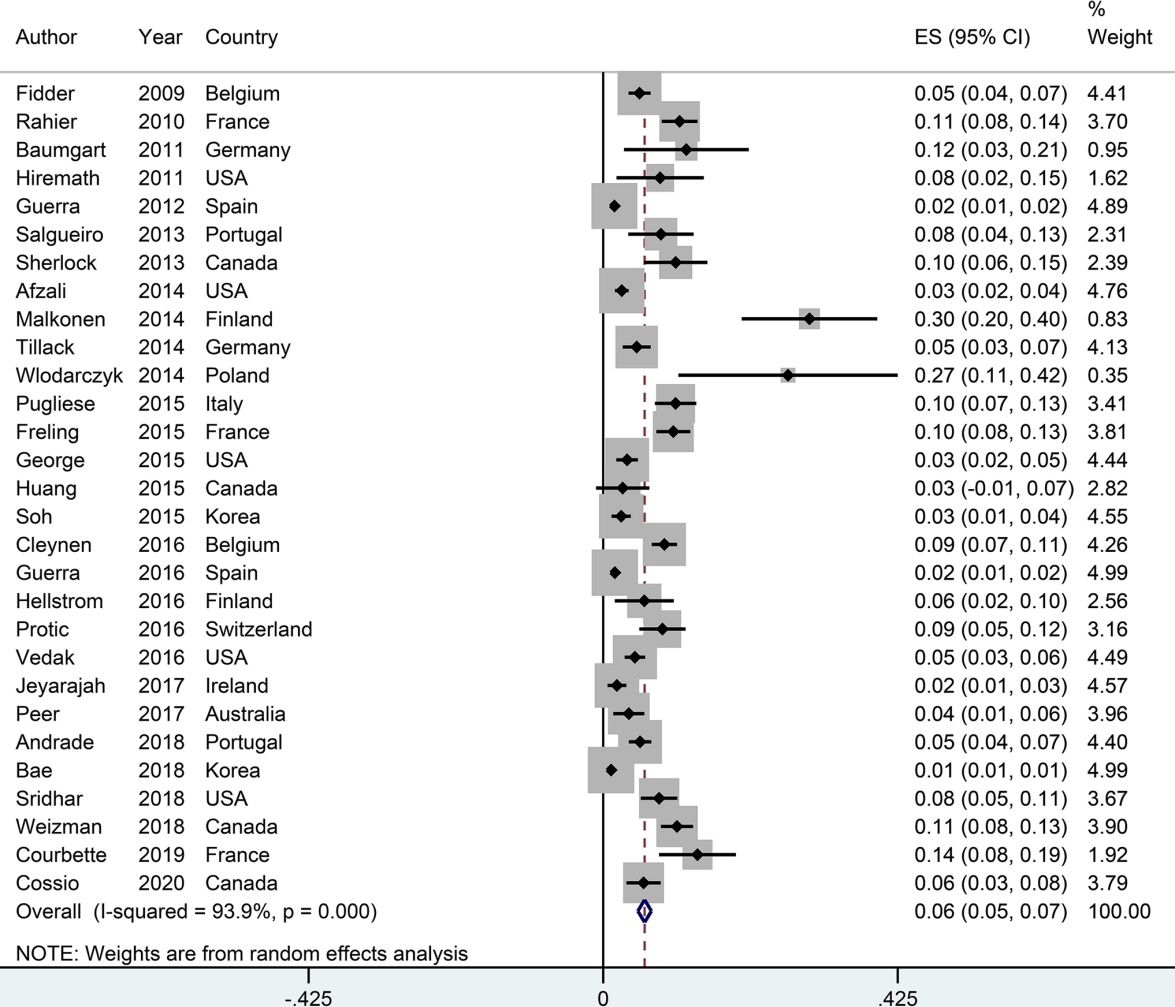
Forest plots of incidence of psoriasiform lesions and/or psoriasis associated with anti-tumor necrosis factor therapy in inflammatory bowel disease patients.

The incidence of psoriasis or psoriasiform lesions associated with anti-TNF therapy in IBD patients was reported by 29 articles, varying from 1.1 to 29.8%. In total, 913 cases were documented from 24,547 patients with IBD exposed to anti-TNF agents, corresponding to a crude incidence of 3.7%. The overall pooled incidence of psoriasis and/or psoriasiform lesions following anti-TNF therapy was 6.0% (95% CI, 5.0–7.0%; *I* ^2^ = 93.9%, *n* = 29 studies), with 6.9% (95% CI 5.1–8.7%; *I* ^2^ = 88.7%, *n* = 15 studies) for psoriasiform lesions and 4.6% (95% CI 3.6–5.6%; *I* ^2^ = 93.3%, *n* = 15 studies) for psoriasis, respectively (**Figures 1** **–** **3**). The results of the jackknife sensitivity analysis suggested that the pooled estimate was robust and not influenced excessively by omitting any single study, ranging from 5.7 to 6.5% (**Supplementary Table S2**). When tested for publication bias, *P* was 0.034 for Begg’s test and 0.001 for Egger’s test. Despite the existence of a publication bias, the sensitivity analyses of the trim-and-filled method showed that the result was roughly reliable. We further conducted a sensitivity analysis exclusively including the 23 studies that reported an incidence of *de novo* psoriasis/psoriasiform lesion, yielding a similar pooled incidence of 5.8% (4.8–6.9%) (6–15, 22, 24, 25, 27, 30–32, 34–38, 40) (**Supplementary Figure S2**).

**Figure 2:**
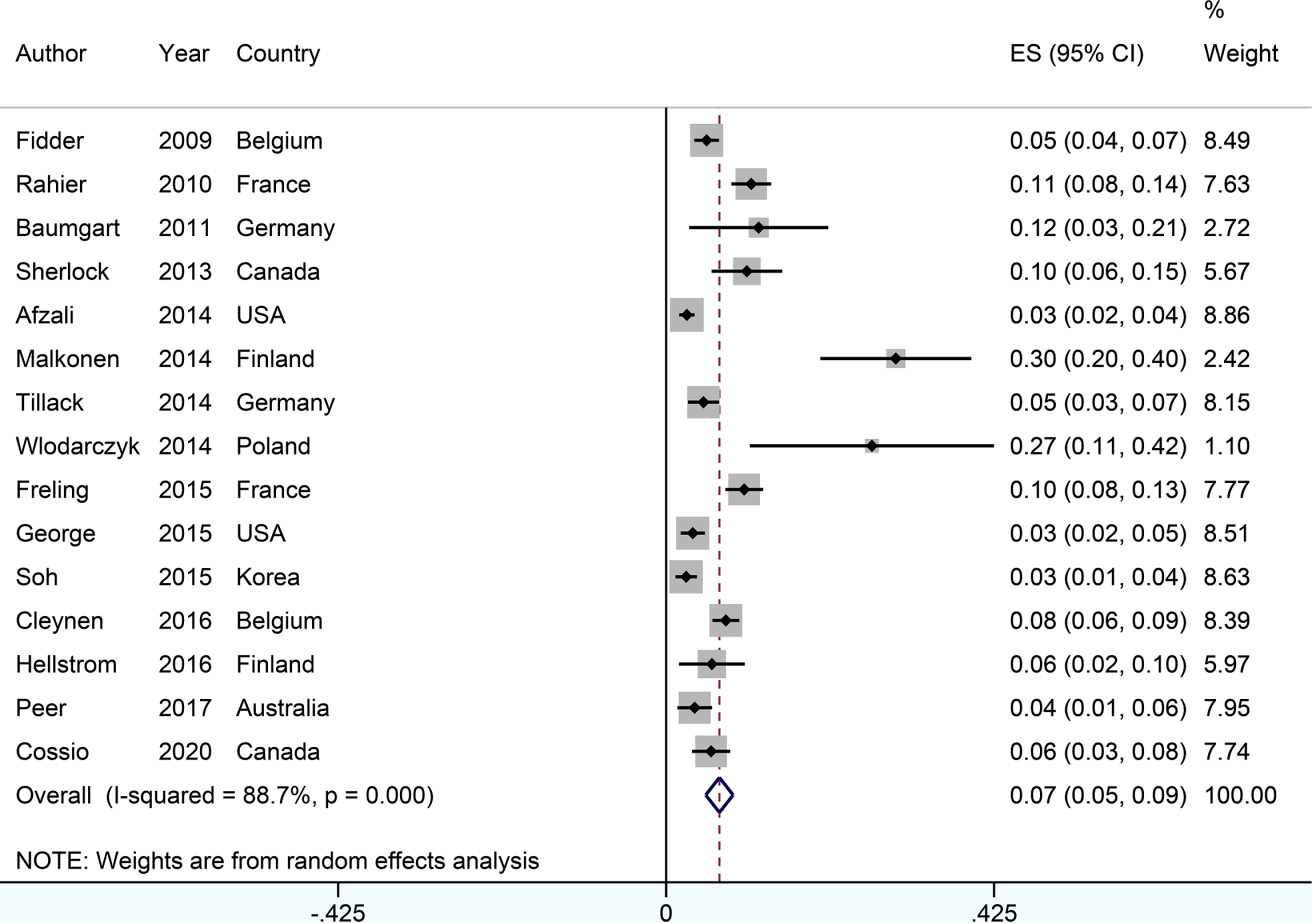
Forest plots of incidence of psoriasiform lesions associated with anti-tumor necrosis factor therapy in inflammatory bowel disease patients.

**Figure 3:**
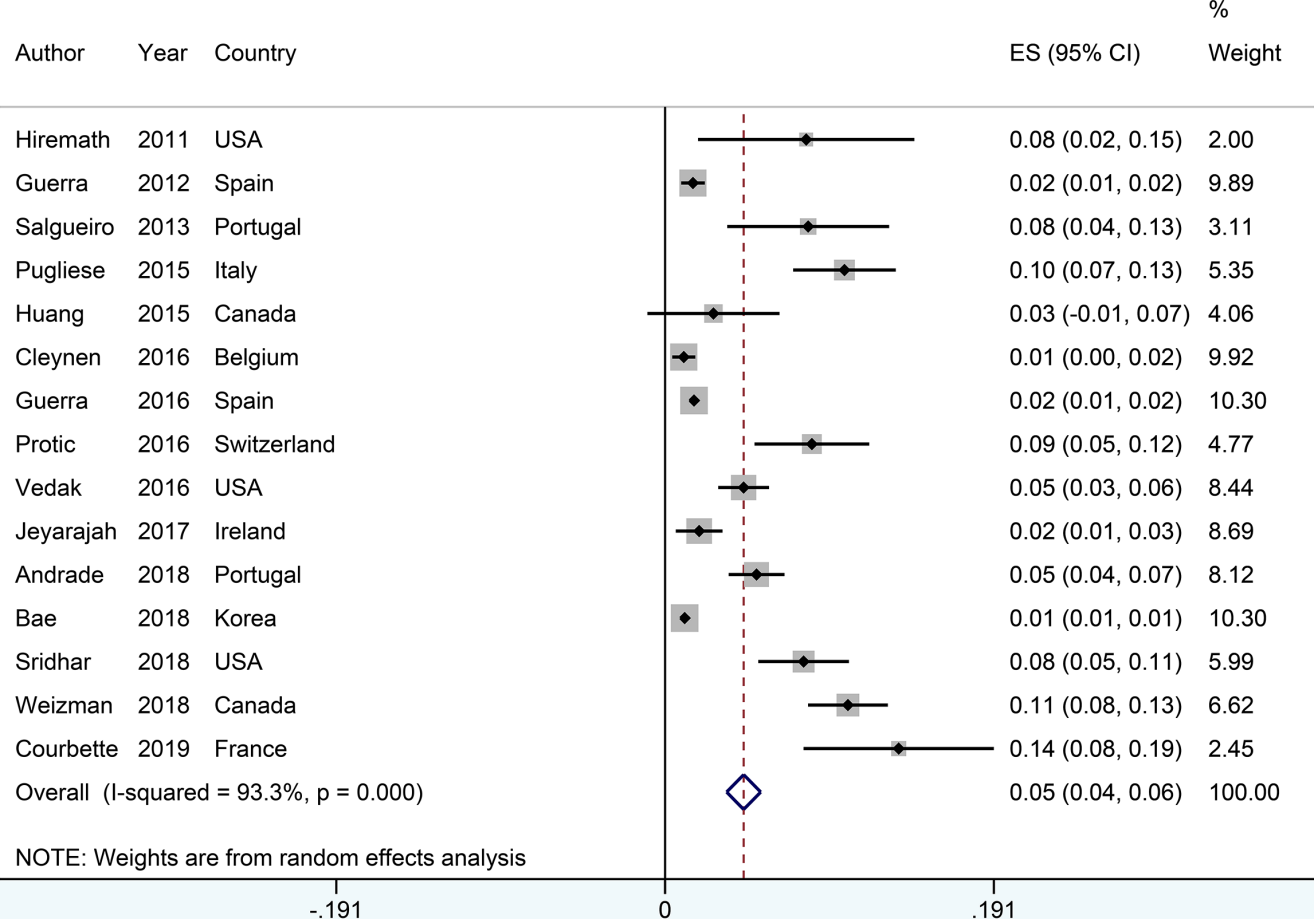
Forest plots of incidence of psoriasis associated with anti-tumor necrosis factor therapy in inflammatory bowel disease patients.

In addition, the pooled incidence of psoriasis and/or psoriasiform reaction was separately analyzed according to gender (5.6%, 95% CI: 3.9–7.2% for female, *n* = 10 studies; 5.6%, 95% CI 3.9–7.2% for male, *n* = 10 studies), the types of IBD [4.8%, 95% CI: 3.7–5.9% for Crohn’s disease (CD), *n* = 14 studies; 3.0%, 95% CI: 1.9–4.1% for ulcerative colitis (UC), *n* = 12 studies], and anti-TNF agents (5.4%, 95% CI: 4.2–6.5% for infliximab, *n* = 15 studies; 4.3%, 95% CI: 2.9–5.6% for adalimumab, *n* = 11 studies; 6.8%, 95% CI: 3.7–9.9% for certolizumab, *n* = 2 studies) (**Supplementary Figures S3**–**S9**).

### Meta−Regression and Subgroup Analysis

Meta-regression analysis was conducted to investigate the source of heterogeneity. Nine covariates, including publication year, setting, study design, region, population, sample size, CD proportion, infliximab proportion, and study quality, were extracted from the 29 included studies. The univariable meta-regression identified three factors potentially related to the heterogeneity, including study design, region, population, and sample size (**Table 2**). As only 29 studies were included, we further included 3 factors in the multivariable meta-regression, showing region (coefficient -0.244, 95% CI: -0.046 to 0.003, *P* = 0.029) and population (coefficient 0.023, 95% CI -0.001 to 0.047, *P* = 0.062) to have significantly contributed to the heterogeneity (**Table 2**).

**Table 2:** Meta-regression for the source of heterogeneity of pooled incidence.

Variables	Number of studies	Coefficient	*P*-value	95% CI	Adjusted *R* ^2^
**Univariate analysis**					
Publication year	29	-0.002	0.575	-0.009, 0.005	-5.53%
Setting	29	-0.035	0.167	-0.085, 0.153	7.25%
Study design	29	0.055	0.066	-0.004, 0.111	-4.33%
Region	29	-0.023	0.073	-0.049, 0.023	10.60%
Population	27	0.0289	0.025	0.004, 0.054	26.52%
Sample size	29	-1.05e-05	0.044	-2.06e-05, -3.01 e-07	20.38%
CD proportion	23	0.012	0.294	-0.011, 0.351	-5.44%
IFX proportion	21	0.037	0.618	-0.116, 0.190	-7.63%
Psoriasis diagnosis	29	-0.016	0.407	-0.057, 0.024	-2.69%
Study outcome	29	-0.017	0.382	-0.058, 0.023	-4.99%
Study quality	29	0.115	0.275	-0.010, 0.033	0.85%
**Multivariate analysis**					
Region	27	-0.244	0.029	-0.046, 0.003	47.63%
Population		0.023	0.062	-0.001, 0.047	
Sample size		-5.66e-06	0.234	-1.52 e-05, 3.92e-06	

We further conducted several subgroup analyses according to region, setting and study design, sample size, diagnosis method, and study quality (**Table 3**). These results indicated that the heterogeneity can also be partially explained by the differences in region and population.

**Table 3:** Subgroup analyses of pooled incidence of psoriasis or psoriasiform lesions associated with anti-tumor necrosis factor therapy in inflammatory bowel disease patients.

Subgroup	Number of studies	Pooled incidence (95% CI)	*I* ^2^
**Region**			
Europe	17	7.4% (5.7–9.1%)	94.6%
North America	9	6.0% (4.0–7.9%)	86.2%
Asia-Pacific	3	2.2% (5.0–7.0%)	77.0%
**Setting**			
Monocenter	25	6.8% (5.5–8.2%)	87.3%
Multicenter	4	2.5% (1.5–3.6%)	95.0%
**Study** D**esign**			
Retrospective	24	5.6% (4.6–6.6%)	94.3%
Prospective	5	13.7% (6.2–21.2%)	87.5%
**Population**			
Children	6	11.01% (7.0–15.1%)	81.3%
Adult	12	6.6% (4.8–8.4%)	86.9%
Mixed	9	3.7% (2.6–4.7%)	93.3%
**Sample size**			
<500	16	8.2% (5.9–10.2%)	84.2%
≥500	13	4.9% (3.8–6.0%)	95.8%
**Diagnosis method**			
Gastroenterologist/dermatologist	15	5.6% (4.1–7.2%)	91.3%
Not reported	14	6.4% (5.0–7.7%)	94.1%
**Newcastle**–**Ottawa** S**cale score**			
<7	19	4.9% (3.9–5.9%)	91.8%
≥7	10	7.8% (5.5–10.1%)	91.0%

### Risk Factors for Psoriasis or Psoriasiform Lesions

**Figure 4:**
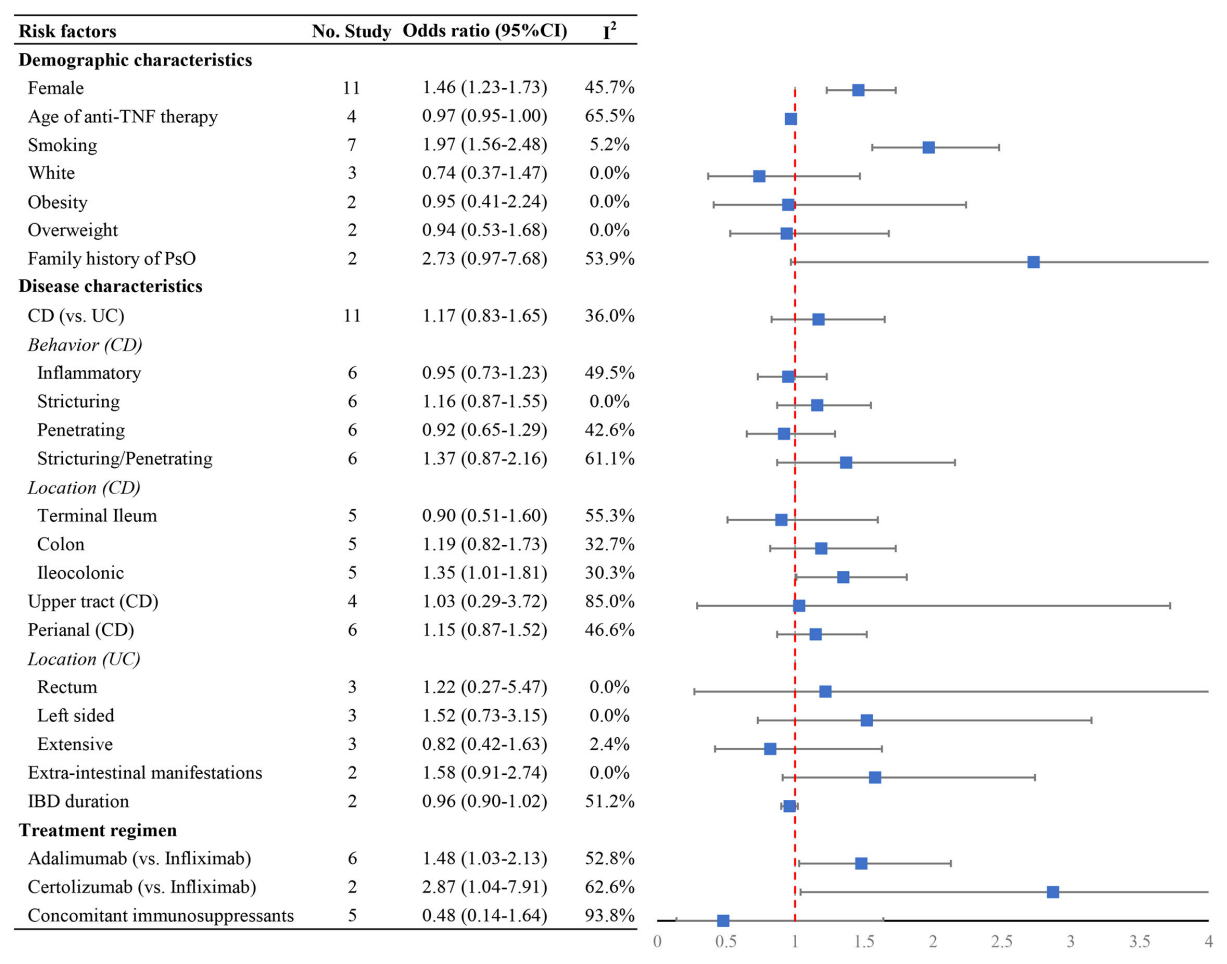
Forest plots of the odds ratio for the risk of developing psoriasiform lesions and/or psoriasis in inflammatory bowel disease patients receiving anti-tumor necrosis factor therapy.

Data on clinical associations of psoriasis or psoriasiform lesions following anti-TNF therapy were available from 18 studies (6, 8, 9, 11–16, 25, 26, 30, 32–34, 36, 38, 40). With regard to demographic features, the risk of psoriasis or psoriasiform lesions during anti-TNF therapy was significantly higher in female patients (pooled OR: 1.46, 95% CI: 1.23–1.73, *I* ^2^ = 45.7%, *n* = 11 studies), with younger age at anti-TNF initiation (pooled OR: 1.03, 95% CI: 1.00–1.05, *I* ^2^ = 65.5%, *n* = 4 studies), and smoking (pooled OR: 1.97, 95% CI: 1.56–2.48, *I* ^2^ = 5.2%, *n* = 7 studies) (**Figure 4** and **Supplementary Figures S10**–**S12**). In the study of Fréling et al., IBD patients aged <28 years had a significantly higher risk of developing psoriasiform lesions compared with those >46 years (hazard ratio: 5.21, 95% CI: 2.43–11.16). No significant association was detected regarding white race, obesity/overweight, and family history of psoriasis (**Figure 4** and **Supplementary Figures S13**–**S16**).

In relation to combinable disease characteristics and treatment regimen, the presence of psoriasis or psoriasiform lesions following anti-TNF exposure was significantly higher in IBD patients receiving adalimumab (OR: 1.48, 95% CI: 1.03–2.13, *I* ^2^ = 52.8%, *n* = 6 studies) and certolizumab (OR: 2.87, 95% CI: 1.04–7.91, *I* ^2^ = 62.6%, *n* = 2 studies) than that of infliximab, but not associated with CD (*versus* UC), longer IBD duration, the presence of extraintestinal manifestations, and concomitant immunosuppressive therapy (**Figure 4** and **Supplementary Figures S17**–**S34**). Moreover, no significant differences were observed regarding disease behavior and location of CD and the extent of UC, except ileocolonic Crohn’s disease with OR of 1.48 (1.03–2.13, *I* ^2^ = 30.3%, *n* = 5 studies) (**Figure 4** and **Supplementary Figures S17**–**S34**). The result of Egger’s and Begg’s tests showed no publication bias for the above-mentioned analyses (**Supplementary Table S3**).

In addition, other factors were included only in the systematic review rather than in the meta-analysis (**Supplementary Table S4**) because the assessment was performed only in 1 study. In this systematic review, no significant associations with psoriasis or psoriasiform lesions in IBD patients receiving anti-TNF therapy were observed, except acute psychological stressor (OR: 3.14, 95% CI: 1.10–8.93) (16) and body mass index (OR: 1.12, 95% CI: 1.01–1.24) (30).

## Discussion

To our knowledge, this is the first systematic review and meta-analysis to comprehensively investigate the incidence of and risk factors for psoriasis or psoriasiform lesions secondary to anti-TNF therapy in IBD patients. The overall estimated pooled incidence was 6.0% psoriasis and/or psoriasiform lesions in IBD patients following anti-TNF therapy. Regarding risk factors, female, younger age at anti-TNF therapy initiation, smoking, and adalimumab or certolizumab usage were significantly associated with an increased risk of developing psoriasis or psoriasiform lesions during anti-TNF therapy in IBD patients. These findings have the potential to inform clinical practice for more individualized decisions or precautions and may help us to understand the mechanism of this paradoxical phenomenon.

Anti-TNF agents have assumed the dominant position in the treatment of IBD over the past couple of decades. A large body of evidence confirms the overall good safety profile of the anti-TNF agents. However, with the increased use of these agents, paradoxical inflammation or autoimmune diseases induced by anti-TNF agents have been continuously reported, including cutaneous, articular, ocular, and neurological involvements (41, 42). Of these, paradoxical psoriasis or psoriasiform lesions, being the most prevalent and well-known paradoxical adverse events associated with anti-TNF agents, have been under intense investigation in recent years. At present, paradoxical psoriasis or psoriasiform lesions can no longer be considered rare in clinical routine, with incidence estimates of greater than 20% with the use of anti-TNF agents in some research. For the incidence of psoriasis/psoriasiform rash, our results, overall, were comparable to the results from the 2021 meta-analysis (43). The primary outcome of dermatological reactions in IBD patients receiving anti-TNF therapy from 26 studies was 19.4% (95% CI: 15.2–24.4%, *I* ^2^ = 95%) in this meta-analysis. In the secondary outcome of psoriasis/psoriasiform rash, there was a pooled incidence of 5.6% (95% CI: 4.2–7.4%, *I* ^2^ = 95%), with 6.1% (95% CI: 3.4–10.6%, *I* ^2^ = 96%) for infliximab therapy and 5.9% (95% CI: 2.5–13.5, *I* ^2^ = 93%) for adalimumab therapy. In addition to this, our work has reported more detailed and more specific information on anti-TNF associated psoriasis/psoriasiform rash. In the meta-regression and subgroup analyses, we also noticed that the regions and population contributed to the heterogeneity. Taken together, gastroenterologists should be aware of the paradoxical phenomenon, and the current findings could be instrumental in guiding therapeutic decision in clinical routine.

Currently, the molecular mechanisms and pathogenesis of paradoxical psoriasis/psoriasiform rash associated with anti-TNF agents are poorly understood, and multiple factors might be involved, including the genetic predisposition, preexisting autoimmune condition, and increased secretion and imbalance of cytokines and cells (interferon−α, Th1, Th2, Th17 cytokines, *etc.*). Clinically, the risk factors for developing psoriasis/psoriasiform rash after anti-TNF therapy are under exploration but are inconclusive. The present study, for the first time, has systematically reviewed the literature surrounding the risk factors. The meta-analyses revealed a statistically increased risk of developing psoriasis or psoriasiform lesions during anti-TNF therapy in IBD patients who are female, of a young age at anti-TNF therapy initiation, smoking, and using adalimumab or certolizumab. In the general population, psoriasis can manifest at any age, but with the highest peak between the ages of 20 and 40 years (44). The function of the immune system, and so does autoimmunity, is affected by various factors, including age (45). Overall, age is closely related to the strength of the immune system response, which is expected to decline in senescence (46, 47). From this aspect, the association between advanced age and low risk of psoriasis or psoriasiform lesions secondary to anti-TNF therapy can also be, in part, instinctively understood. In this study, we found that smoking, past and present, is the major risk factor for developing psoriasis during anti-TNF treatment in IBD patients. In fact, the adverse effects of smoking on psoriasis onset have been documented in the general population. The possible pathophysiological mechanisms of the associations included oxidative stress and free radical damage induced by smoking, which could trigger a cascade of systemic inflammation and the subsequent development of psoriasis (48, 49). However, it is still challenging to understand whether or how smoking work together with TNF blockade to orchestrate the psoriasis occurrence. *In vitro*, cigarette smoke chemical components could activate nuclear factor kappa-B activation and proinflammatory cytokine production, including IL-1β and IL-6 (50). The full blockade of TNF-α may impair the homeostasis of normal skin and cause an imbalance in cytokines and cells, which may be further exacerbated by the presence of smoking, and finally paradoxical adverse events occur (50). For paradoxical skin inflammation, the IFN-α pathway was considered to play a key role. However, cigarette smoking was found to decrease the production of IFN-α and increase the production of IFN-β *in vitro* (51). Unraveling the synergistic effect between smoking and TNF blockade on the incidence of paradoxical psoriasis can be extremely complex in people with IBD, yet smoking cessation before starting anti-TNF therapy merits consideration in IBD patients from the perspective of decreasing the risk of paradoxical adverse events. In addition, current evidence suggests that paradoxical inflammation during treatment with anti-TNF agents seems to be a drug class effect. In the present study, the significantly higher risk of adalimumab or certolizumab therapy than infliximab therapy was identified, although both of them were associated with an increased risk of paradoxical psoriasis or psoriasiform skin lesions. In fact, potential differences between adalimumab and infliximab in IBD have been reported. In a nationwide cohort study of biologic-naive adults with UC, the adalimumab-treated patients showed a substantially higher rate of all-cause hospitalization and serious infection requiring hospitalization and a trend toward a higher rate of UC-related hospitalization (52). Besides these, infliximab drug levels were found to be associated with the depth of remission in patients with CD, but no such relationship was detected for adalimumab (53). However, there is no plausible mechanism evidence to explicitly explain the difference among different types of anti-TNF-associated psoriasis or psoriasiform rash. Future clinical and basic science studies are needed to experimentally validate the presented findings.

However, there were several limitations to our study. First, due to the nature of observational design in the original studies, the present study is vulnerable to potential biases (information or selection bias), which cannot allow us to conclude definite causal relationships. Second, the heterogeneity for the pooled incidence among the studies was very high. For this, we performed a series of subgroup analyses, meta-regression, and risk factor exploration. To a large extent, they could explain the source of heterogeneity. Third, various diagnosis criteria of psoriasis or psoriasiform skin lesions were applied in the included studies, mostly by interview or read codes rather than by dermatologists. Establishing a close collaboration between gastroenterologists and dermatologists is necessary to overcome this limitation in the future. Fourth, not all studies made enough adjustment for potential confounders, and we cannot fully unify the confounders, which can potentially lead to either an overestimation or an underestimation of the associations. Lastly, despite all the potential risk factors evaluated, for some of them, especially for disease activity, cumulative anti-TNF dosages were only included into the systematical review, and further investigations are required to explore their association.

## Conclusion

In summary, the overall estimated pooled incidence of psoriasis/psoriasiform lesions secondary to anti-TNF therapy was 6% in IBD patients. Female, young age at anti-TNF therapy initiation, smoking, ileocolonic CD, and adalimumab or certolizumab use were associated with a substantially increased risk of developing psoriasis or psoriasiform lesions during anti-TNF therapy. These findings have the potential to inform clinical practice for more individualized decisions or precautions and may help us to understand the mechanism of this paradoxical phenomenon.

## Data Availability Statement

The original contributions presented in the study are included in the article/**Supplementary Material**. Further inquiries can be directed to the corresponding author.

## Author Contributions

ZZ conceptualized the study, participated in its design and coordination, and critically revised the manuscript. WX and SX contributed to data collection, analysis, and interpretation and drafted the manuscript. HH contributed to the process of data collection as a study investigator. All authors contributed to the article and approved the submitted version.

## Conflict of Interest

The authors declare that the research was conducted in the absence of any commercial or financial relationships that could be construed as a potential conflict of interest.

## Publisher’s Note

All claims expressed in this article are solely those of the authors and do not necessarily represent those of their affiliated organizations, or those of the publisher, the editors and the reviewers. Any product that may be evaluated in this article, or claim that may be made by its manufacturer, is not guaranteed or endorsed by the publisher.
