# Supplementary material for: Incidence of and Risk Factors for Paradoxical Psoriasis or Psoriasiform Lesions in Inflammatory Bowel Disease Patients Receiving Anti-TNF Therapy: Systematic Review With Meta-Analysis
Source: Front Immunol. 2022 Mar 1;13:847160. doi: 10.3389/fimmu.2022.847160 (PMC8921985; doi:10.3389/fimmu.2022.847160)
Supplement: Supplementary file 1 [file DataSheet_1.docx]

**Supplementary File**

Additional Supporting Information may be found in the online version of this article at the publisher’s website.

**SUPPLEMENTARY APPENDIX**

**Appendix S1.** Search strategy.

**Table S1**. Quality assessment of cohort and case-control studies using the Newcastle-Ottawa scale

**Table S2.** Sensitivity analysis using the jackknife approach

**Table S3.** Egger’s test and Begger’s test for publication bias.

**Table S4.** The summary of other risk factors in included studies.

**Figure S1.** PRISMA flow diagram of study selection for systematic review and meta-analysis. PRISMA: Preferred Reporting Items for Systematic Reviews and Meta-Analyses.

**Figure S2.** Sensitivity analysis exclusively including the studies that reported incidence of de novo psoriasis/psoriasiform lesion.

**Figure S3.** Forest plots of incidence of psoriasiform lesions and/or psoriasis associated with anti-TNF therapy in female IBD patients

**Figure S4.** Forest plots of incidence of psoriasiform lesions and/or psoriasis associated with anti-TNF therapy in male IBD patients

**Figure S5.** Forest plots of incidence of psoriasiform lesions and/or psoriasis associated with anti-TNF therapy in CD patients

**Figure S6.** Forest plots of incidence of psoriasiform lesions and/or psoriasis associated with anti-TNF therapy in UC patients

**Figure S7.** Forest plots of incidence of psoriasiform lesions and/or psoriasis associated with infliximab therapy in IBD patients

**Figure S8.** Forest plots of incidence of psoriasiform lesions and/or psoriasis associated with adalimumab therapy in IBD patients.

**Figure S9.** Forest plots of incidence of psoriasiform lesions and/or psoriasis associated with certolizumab therapy in IBD patients

**Figure S10.** Forest plots of the odds ratio for female

**Figure S11.** Forest plots of the odds ratio for age of anti-TNF therapy

**Figure S12.** Forest plots of the odds ratio for smoking

**Figure S13.** Forest plots of the odds ratio for white

**Figure S14.** Forest plots of the odds ratio for obesity

**Figure S15.** Forest plots of the odds ratio for overweight

**Figure S16.** Forest plots of the odds ratio for family history of PsO

**Figure S17.** Forest plots of the odds ratio for CD (vs. UC)

**Figure S18.** Forest plots of the odds ratio for inflammatory CD

**Figure S19.** Forest plots of the odds ratio for stricturing CD

**Figure S20.** Forest plots of the odds ratio for penetrating CD

**Figure S21.** Forest plots of the odds ratio for stricturing/penetrating CD

**Figure S22.** Forest plots of the odds ratio for terminal Ileum CD

**Figure S23.** Forest plots of the odds ratio for colon CD

**Figure S24.** Forest plots of the odds ratio for ileocolonic CD

**Figure S25.** Forest plots of the odds ratio for upper tract CD

**Figure S26.** Forest plots of the odds ratio for perianal CD

**Figure S27.** Forest plots of the odds ratio for rectum UC

**Figure S28.** Forest plots of the odds ratio for left sided UC

**Figure S29.** Forest plots of the odds ratio for extensive UC

**Figure S30.** Forest plots of the odds ratio for extra-intestinal manifestations

**Figure S31.** Forest plots of the odds ratio for IBD duration

**Figure S32.** Forest plots of the odds ratio for adalimumab (vs. infliximab).

**Figure S33.** Forest plots of the odds ratio for certolizumab (vs. infliximab)

**Figure S34.** Forest plots of the odds ratio for concomitant immunosuppressants

**Appendix S1**

**Search strategy:**

(inflammatory bowel disease* OR Crohn’s disease OR Crohn disease OR ulcerative colitis) and (psoriasis OR psoriasiform OR dermatological OR skin OR cutaneous) and (anti-tumor necrosis factor alpha OR anti-tumor necrosis factor-α OR anti-TNFα OR anti-TNF OR tumor necrosis factor alpha antagonist* OR tumor necrosis factor-α antagonist* OR tumor necrosis factor antagonist* OR TNF-a antagonist* OR TNF antagonist* OR tumor necrosis factor alpha inhibitor* OR tumor necrosis factor-α inhibitor* OR tumor necrosis factor inhibitor* OR TNFi OR infliximab OR Remicade or adalimumab OR Humira OR certolizumab OR Cimzia OR golimumab OR Simponi)

**Table S1**. Quality assessment of cohort and case-control studies using the Newcastle-Ottawa scale

| Study | Selection^&^ | | | | Comparability* | Outcome^#^ | | | Total |
| --- | --- | --- | --- | --- | --- | --- | --- | --- | --- |
|  | Representativeness  of exposed cohort | Selection of  nonexposed  cohort | Ascertainment  of exposure | Demonstration  outcome not  present at  study start | Comparability  of cohorts | Assessment  of outcome | Follow-up  enough for  outcomes to occur | Adequacy  Of followup |  |
| Fidder | 1 | 1 | 1 | 1 | 2 | 1 | 1 | 0 | 8 |
| Rahier | 1 | 1 | 0 | 1 | 2 | 0 | 1 | 0 | 6 |
| Baumgart | 1 | 1 | 0 | 1 | 2 | 0 | 1 | 0 | 6 |
| Hiremath | 1 | 1 | 0 | 1 | 2 | 0 | 0 | 0 | 5 |
| Guerra | 1 | 1 | 0 | 1 | 2 | 1 | 0 | 0 | 6 |
| Salgueiro | 1 | 1 | 0 | 1 | 2 | 0 | 0 | 0 | 5 |
| Sherlock | 1 | 1 | 0 | 1 | 2 | 0 | 1 | 0 | 6 |
| Afzali | 1 | 1 | 0 | 1 | 2 | 1 | 0 | 0 | 6 |
| Malkonen | 1 | 1 | 1 | 1 | 2 | 0 | 1 | 0 | 7 |
| Tillack | 1 | 1 | 1 | 1 | 2 | 1 | 0 | 0 | 7 |
| Wlodarczyk | 0 | 1 | 0 | 1 | 2 | 1 | 1 | 0 | 6 |
| Pugliese | 1 | 1 | 0 | 1 | 2 | 0 | 1 | 0 | 6 |
| Freling | 1 | 1 | 0 | 1 | 2 | 1 | 1 | 0 | 7 |
| George | 1 | 1 | 1 | 1 | 2 | 1 | 0 | 0 | 7 |
| Huang | 1 | 1 | 0 | 1 | 2 | 1 | 0 | 0 | 6 |
| Soh | 1 | 1 | 1 | 1 | 2 | 1 | 0 | 0 | 7 |
| Cleynen | 1 | 1 | 0 | 1 | 2 | 1 | 1 | 0 | 7 |
| Guerra | 1 | 1 | 0 | 1 | 2 | 0 | 0 | 0 | 5 |
| Hellstrom | 1 | 1 | 0 | 1 | 2 | 0 | 0 | 0 | 5 |
| Protic | 1 | 1 | 0 | 1 | 2 | 0 | 0 | 0 | 5 |
| Vedak | 1 | 1 | 0 | 1 | 2 | 1 | 0 | 0 | 6 |
| Jeyarajah | 1 | 1 | 0 | 1 | 2 | 0 | 0 | 0 | 5 |
| Peer | 1 | 1 | 0 | 1 | 2 | 1 | 0 | 0 | 6 |
| Andrade | 1 | 1 | 0 | 1 | 2 | 0 | 0 | 0 | 5 |
| Bae | 1 | 1 | 0 | 1 | 2 | 0 | 0 | 0 | 5 |
| Sridhar | 1 | 1 | 0 | 1 | 2 | 0 | 0 | 0 | 5 |
| Weizman | 1 | 1 | 0 | 1 | 2 | 1 | 1 | 0 | 7 |
| Courbette | 1 | 1 | 0 | 1 | 2 | 1 | 1 | 0 | 7 |
| Cossio | 1 | 1 | 1 | 1 | 2 | 1 | 0 | 0 | 7 |
| Ya | 1 | 1 | 1 | 1 | 2 | 1 | 0 | 0 | 7 |

&Representativeness of the exposed cohort:

1: Given if representative of the average patient with IBD in the community or patients were consecutively enrolled.

0: Given if selected from a group of volunteers or derivation of the cohort is not described.

&Selection of the nonexposed cohort:

1: Given if drawn from the same community as the exposed cohort.

0: Given if selected from a group of volunteers or derivation of the cohort is not described.

&Ascertainment of exposure

1: Given if obtained by a secure record or structured interview.

0: Given if no description is given or self-report.

&Demonstration that outcome was not present at start of study:

1: Given if demonstrated.

0: Given if not demonstrated.

*Comparability of cohorts on the basis of design or analysis

2: Given if the general baseline characteristics were comparable, including age, gender, disease duration, etc.

0: Given if not demonstrated.

^#^ Assessment of outcome:

1: Given if obtained by patient and/or physician interview and medical record review, diagnosis code.

(pathology reports), or National Death record linkage.

2: Given if obtained from self-report or not described.

^#^ Was follow-up long enough for outcomes to occur:

1: Given if follow-up was long than 1 year for outcome to occur or the incidence ≥10%.

0: Given if follow-up was not long enough and the incidence less than 10%.

^#^ Adequacy of follow-up of cohorts:

1: Given if complete follow-up is provided or ≥90% of follow-up is provided.

**Table S2**. Sensitivity analysis using the jackknife approach

| Author | Year | Incidence (95% CI) | Study excluded, Pooled incidence (95% CI) |
| --- | --- | --- | --- |
| All studies | / | 6.0% (5.0%-7.0%) | Not applicable |
| Fidder | 2009 | 5.2% (3.6%-6.9%) | 6.0% (5.0%-7.0%) |
| Rahier | 2010 | 11.0% (8.4%-13.6%) | 5.7% (4.8%-6.7%) |
| Baumgart | 2011 | 12.0% (3.0%-21.0%) | 5.9% (4.9%-6.9%) |
| Hiremath | 2011 | 8.2% (1.9%-14.5%) | 5.9% (5.0%-6.9%) |
| Guerra | 2012 | 1.6% (0.9%-2.3%) | 6.3% (5.2%-7.3%) |
| Salgueiro | 2013 | 8.3% (3.6%-13.0%) | 5.9% (4.9%-6.9%) |
| Sherlock | 2013 | 10.5% (5.9%-15.0%) | 5.9% (4.9%-6.8%) |
| Afzali | 2014 | 2.7% (1.7%-3.7%) | 6.2% (5.2%-7.2%) |
| Malkonen | 2014 | 29.8% (2.0%-39.5%) | 5.7% (4.8%-6.7%) |
| Tillack | 2014 | 4.8% (2.8%-6.9%) | 6.0% (5.0%-7.0%) |
| Wlodarczyk | 2014 | 26.7% (10.8%-42.5%) | 5.9% (4.9%-6.9%) |
| Pugliese | 2015 | 10.4% (7.5%-13.4%) | 5.8% (4.8%-6.8%) |
| Freling | 2015 | 10.1% (7.7%-12.6%) | 5.8% (4.8%-6.7%) |
| George | 2015 | 3.5% (1.9%-5.0%) | 6.1% (5.1%-7.1%) |
| Huang | 2015 | 2.8% (-1.0%-6.7%) | 6.1% (5.1%-7.1%) |
| Soh | 2015 | 2.6% (1.2%-4.0%) | 6.2% (5.1%-7.2%) |
| Cleynen | 2016 | 8.8% (7.0%-10.7%) | 5.8% (4.8%-6.7%) |
| Guerra | 2016 | 1.7% (1.4%-20.0%) | 6.2% (5.1%-7.2%) |
| Hellstrom | 2016 | 5.9% (1.7%-10.2%) | 6.0% (5.0%-7.0%) |
| Protic | 2016 | 8.6% (3.6%-11.9%) | 5.9% (4.9%-6.9%) |
| Vedak | 2016 | 4.6% (3.1%-6.1%) | 6.0% (5.0%-7.0%) |
| Jeyarajah | 2017 | 2.0% (0.6%-3.3%) | 6.2% (5.2%-7.2%) |
| Peer | 2017 | 3.7% (1.5%-6.0%) | 6.1% (5.1%-7.1%) |
| Andrade | 2018 | 5.3% (3.7%-7.0%) | 6.0% (5.0%-7.0%) |
| Bae | 2018 | 1.1% (0.9%-1.4%) | 6.5% (5.3%-7.7%) |
| Sridhar | 2018 | 8.1% (5.4%-10.7%) | 5.9% (4.9%-6.9%) |
| Weizman | 2018 | 10.7% (8.3%-13.0%) | 5.7% (4.8%-6.7%) |
| Courbette | 2019 | 13.6% (8.1%-19.1%) | 5.8% (4.8%-6.8%) |
| Cossio | 2020 | 5.8% (3.4%-8.3%) | 6.0% (5.0%-7.0%) |

**Table S3.** Egger’s test and Begger’s test for publication bias.

| Variables | Egger’s test | Begger’s test |
| --- | --- | --- |
| Age | 0.776 | 1.000 |
| Female | 0.842 | 1.000 |
| Smoking | 0.277 | 0.108 |
| White | 0.735 | 1.000 |
| Crohn’s diseases | 0.071 | 0.198 |
| ADA | 0.074 | 0.062 |
| IS | 0.592 | 1.000 |
| Inflammatory | 0.036 | 0.452 |
| Stricturing | 0.012 | 0.024 |
| Penetrating | 0.152 | 0.462 |
| Stricturing/ Penetrating | 0.067 | 0.707 |
| Terminal Ileum | 0.510 | 0.462 |
| Colon | 0.965 | 0.806 |
| Ileocolonic | 0.087 | 0.086 |
| Upper tract (CD) | 0.175 | 0.308 |
| Perianal | 0.236 | 0.133 |
| Proctitis | 0.984 | 1.000 |
| Left sided | 0.378 | 1.000 |
| Extensive | 0.123 | 0.308 |

**Table S4.** The summary of other risk factors in included studies.

| Author | Factors | Effect size (95% CI) |
| --- | --- | --- |
| Tillack et al. | Body mass index | 1.12 (1.01-1.24) |
| Pugliese et al. | Previous exposure to anti-TNF-a | 0.47 (0.50-2.55)?  (wrong original data) |
| Freling et al. | Onset under first anti-TNF agent | 0.54 (0.31-0.94) |
|  | Dosing of the first anti-TNF (High) | 1.43 (0.89-2.30) |
|  | Duration of involved anti-TNF agent at time of psoriasis | 0.99 (0.98-1.00) |
|  | IBD activity at time of psoriasis | 1.83 (0.78-4.27) |
| George et al. | Other autoimmune conditions | 2.20 (0.49-9.81) |
| Courbette et al. | Jejunum and/or 2/3 proxileon, Reasons for starting infliximab, Anoperineal fistulizing disease, Medical treatment  failure, Severe colitis, Ileocaecal valve  stenosis, history of surgical resection | NS |
| Ya et al. | Alcohol | 0.80 (0.40-1.58) |
|  | Illicit drugs | 1.84 (0.42-8.00) |
|  | Positive ANA | 1.51 (0.79-2.89) |
|  | Elevated WBC | 0.94 (0.42-2.12) |
|  | Peripheral eosinophilia | 1.46 (0.56-3.83) |
|  | Skin or soft tissue infection | 0.93 (0.46-1.87) |
|  | Upper respiratory infection | 0.60 (0.10-3.68) |
|  | Acute psychological stressor | 3.14 (1.10-8.93) |
|  | Gastroenteritis | 0.70 (0.38-1.30) |


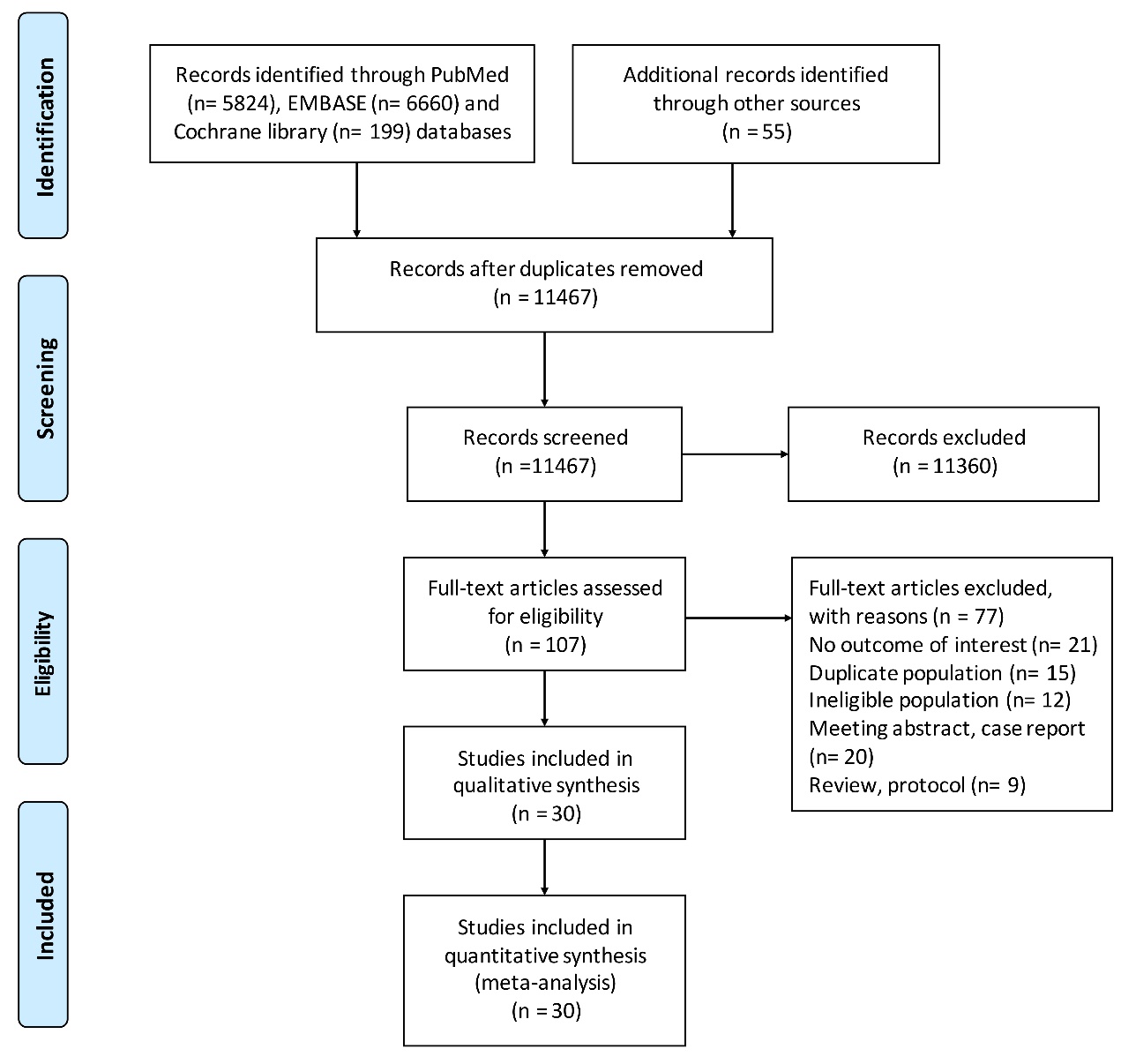
**Figure S1.** PRISMA flow diagram of study selection for systematic review and meta-analysis. PRISMA: Preferred Reporting Items for Systematic Reviews and Meta-Analyses.


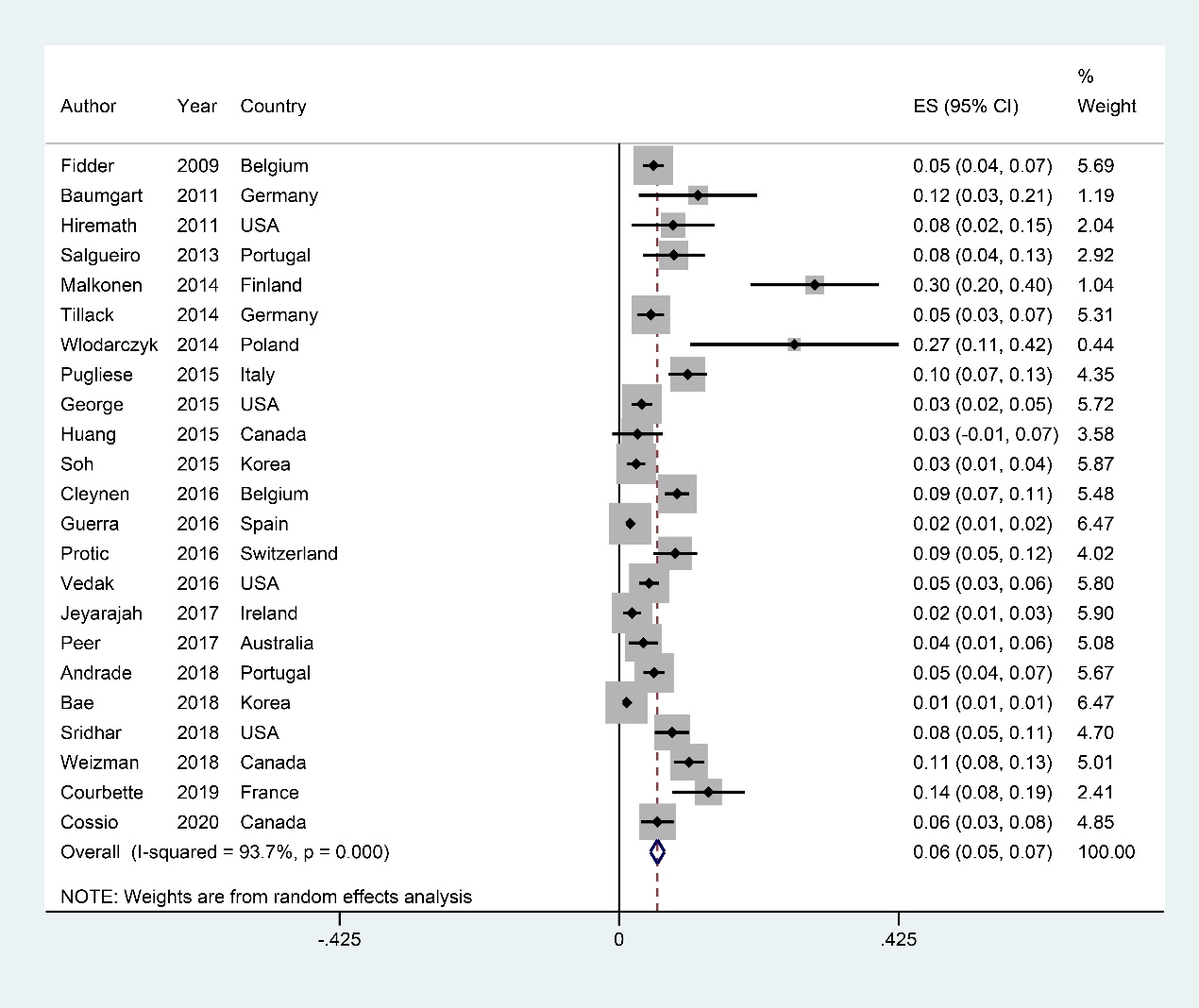
**Figure S2.** Sensitivity analysis exclusively including the studies that reported incidence of de novo psoriasis/psoriasiform lesion.


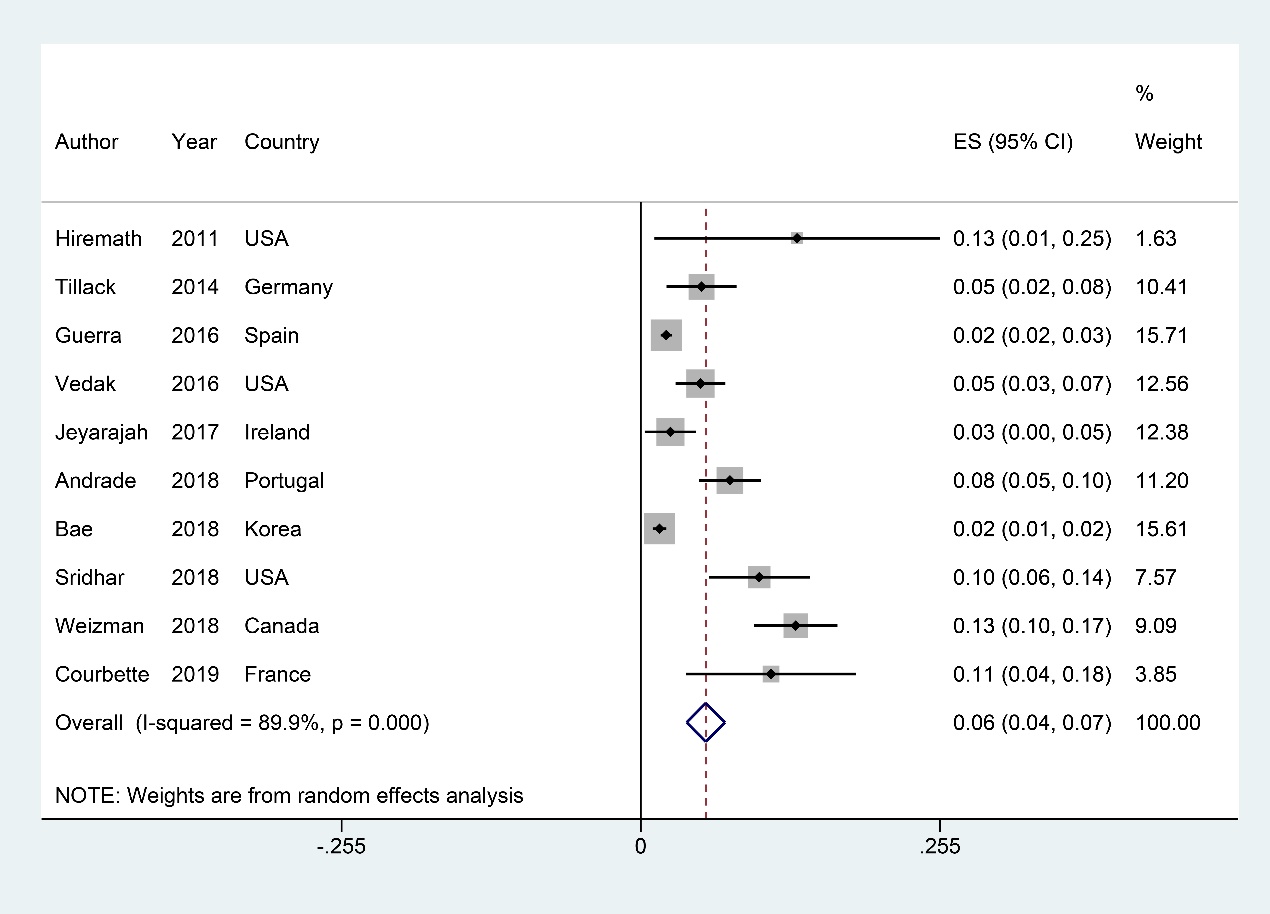
**Figure S3.** Forest plots of incidence of psoriasiform lesions and/or psoriasis associated with anti-TNF therapy in female IBD patients


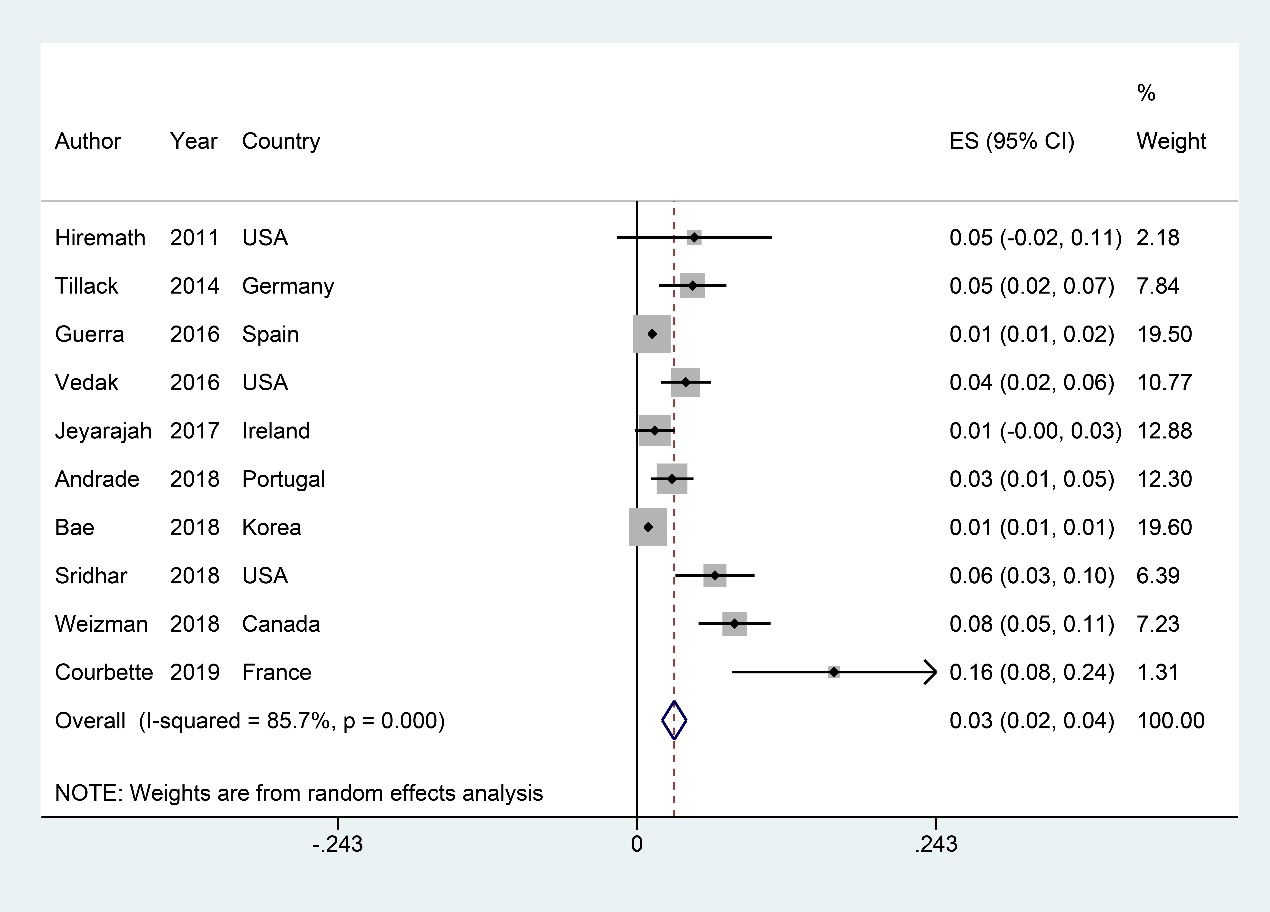
**Figure S4.** Forest plots of incidence of psoriasiform lesions and/or psoriasis associated with anti-TNF therapy in male IBD patients


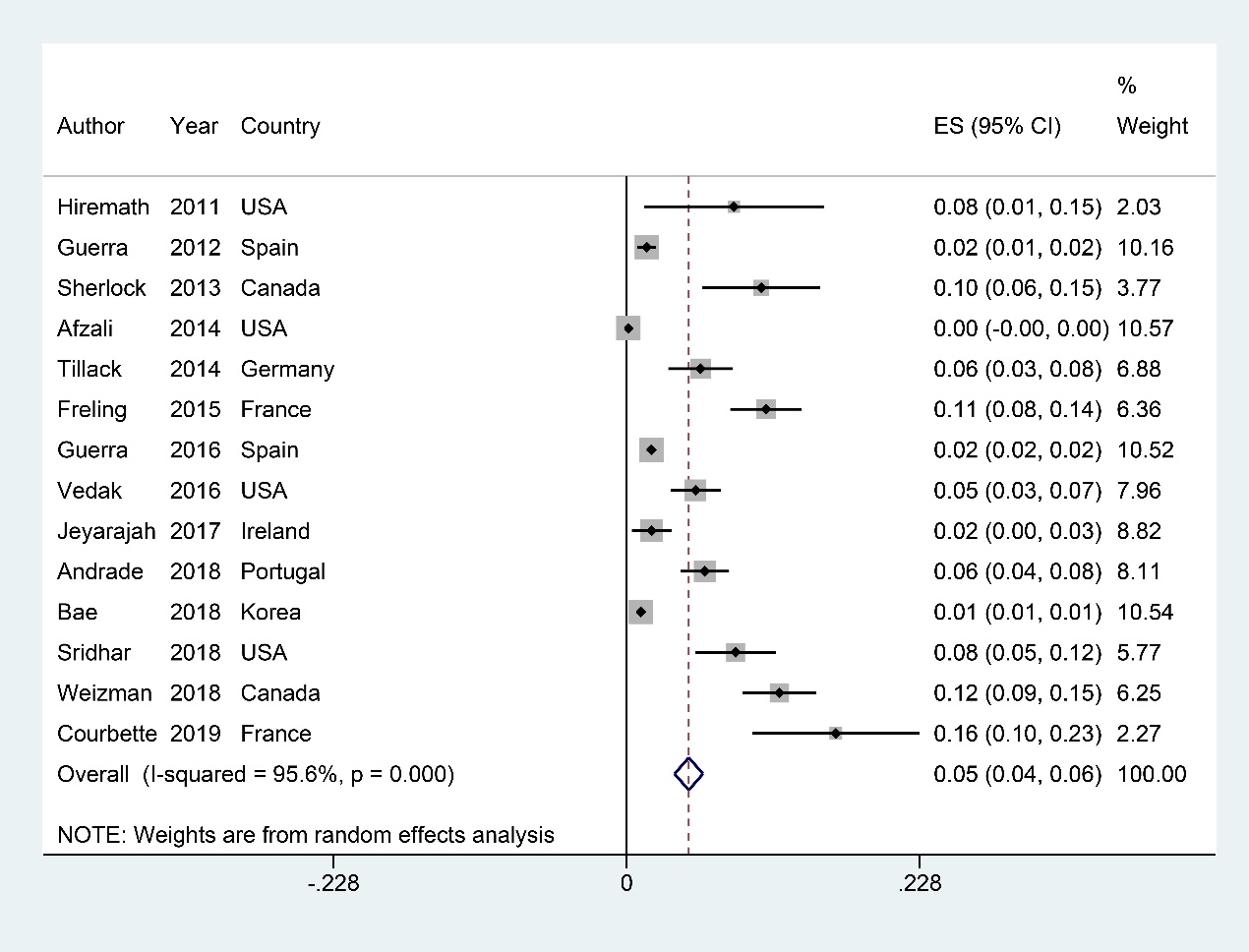
**Figure S5.** Forest plots of incidence of psoriasiform lesions and/or psoriasis associated with anti-TNF therapy in CD patients


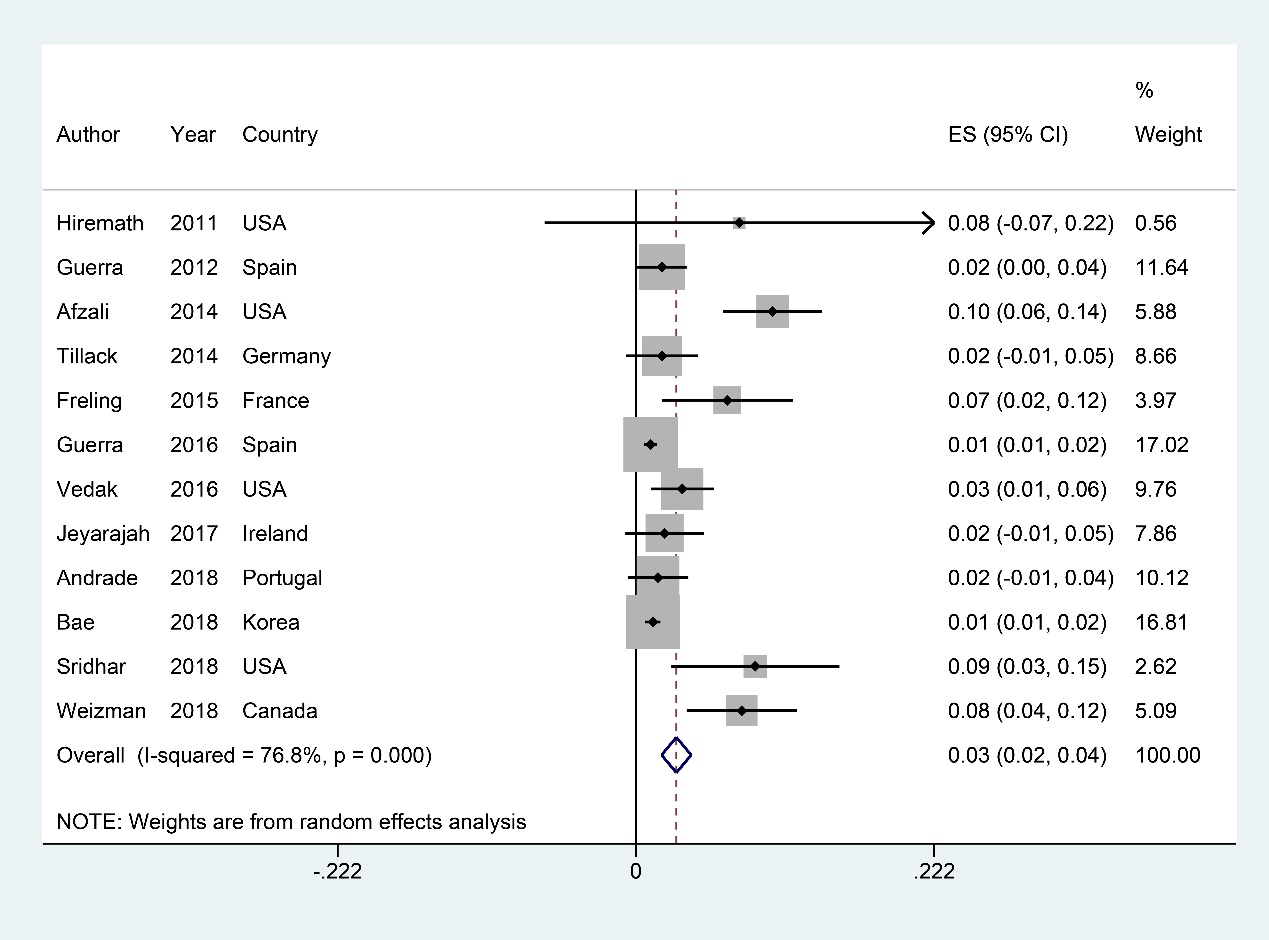
**Figure S6.** Forest plots of incidence of psoriasiform lesions and/or psoriasis associated with anti-TNF therapy in UC patients


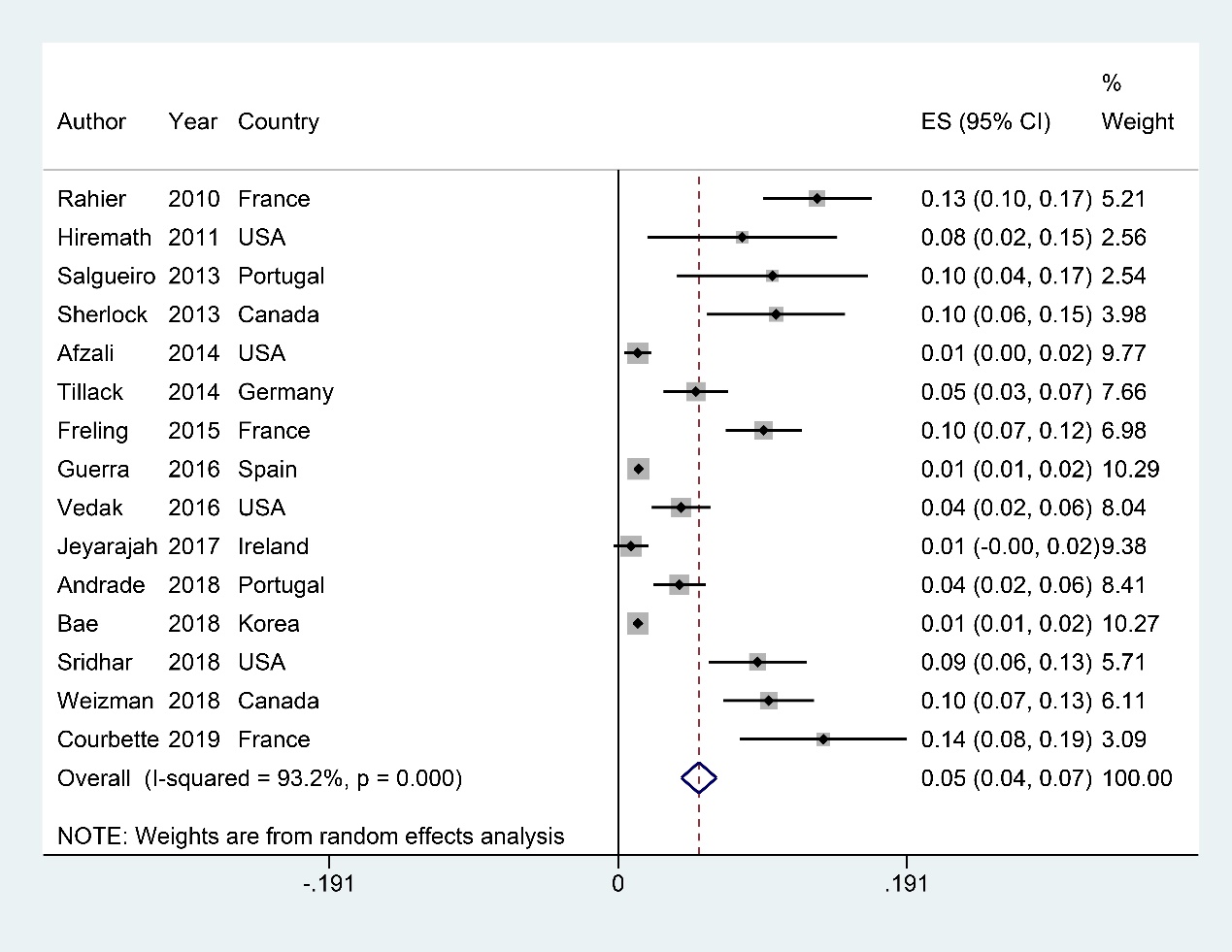
**Figure S7.** Forest plots of incidence of psoriasiform lesions and/or psoriasis associated with infliximab therapy in IBD patients


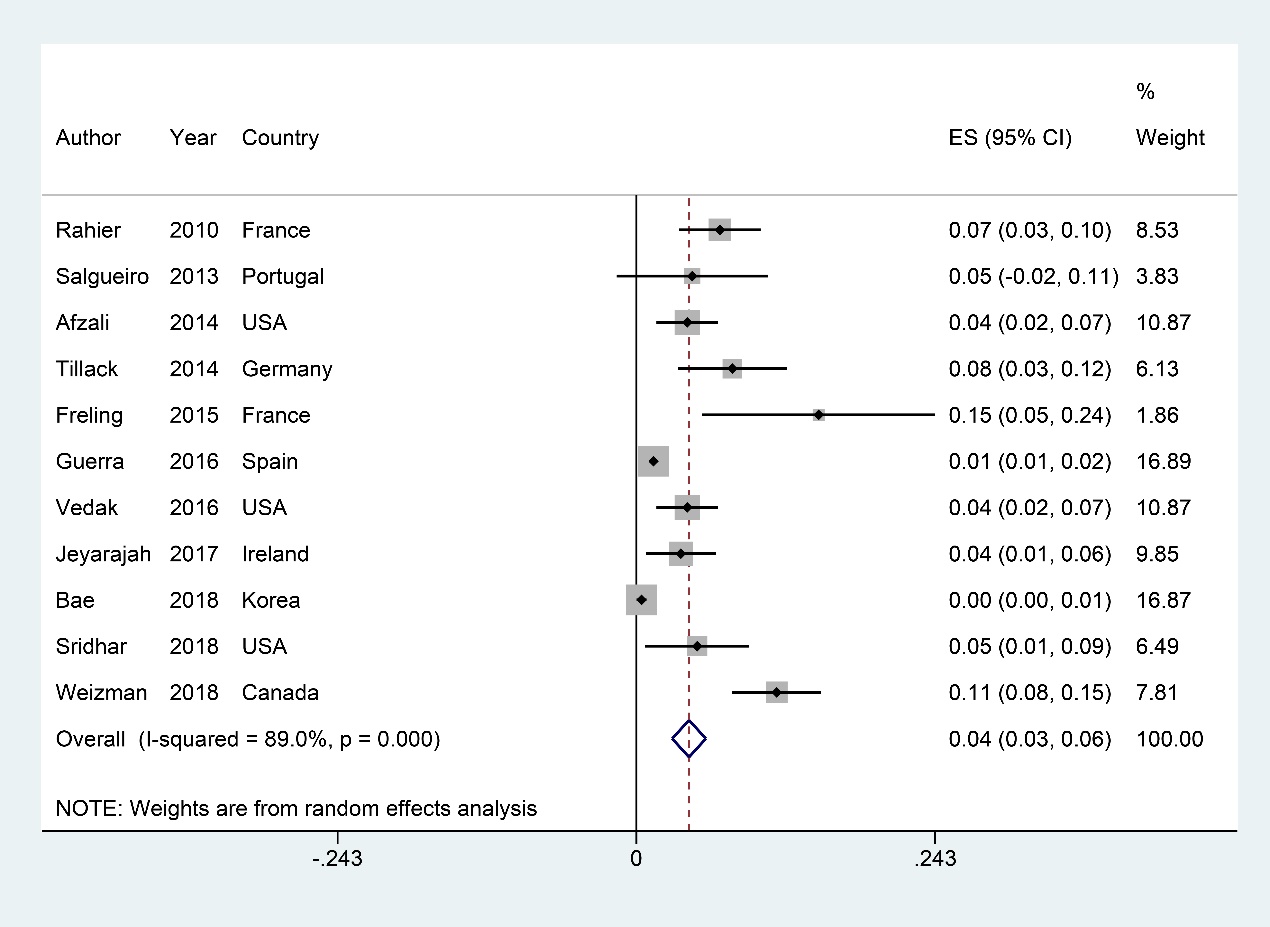
**Figure S8.** Forest plots of incidence of psoriasiform lesions and/or psoriasis associated with adalimumab therapy in IBD patients.


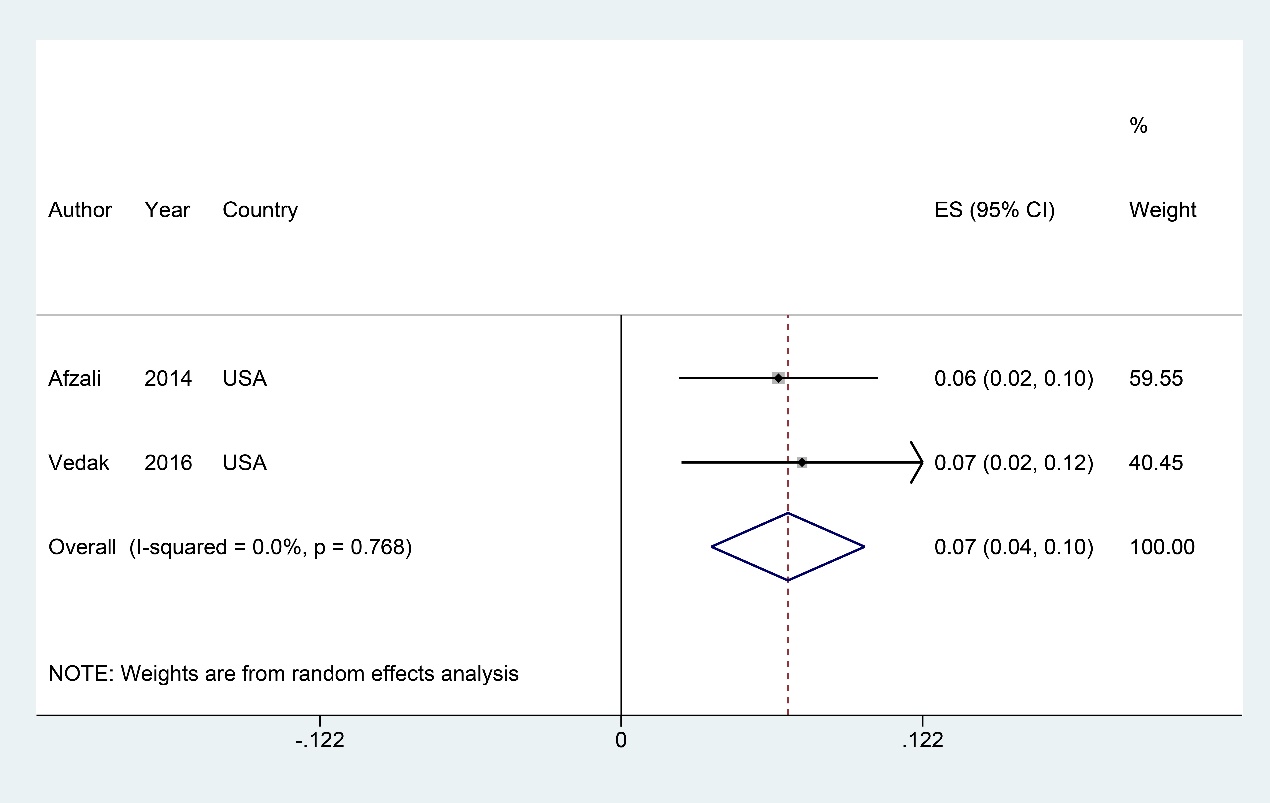
**Figure S9.** Forest plots of incidence of psoriasiform lesions and/or psoriasis associated with certolizumab therapy in IBD patients


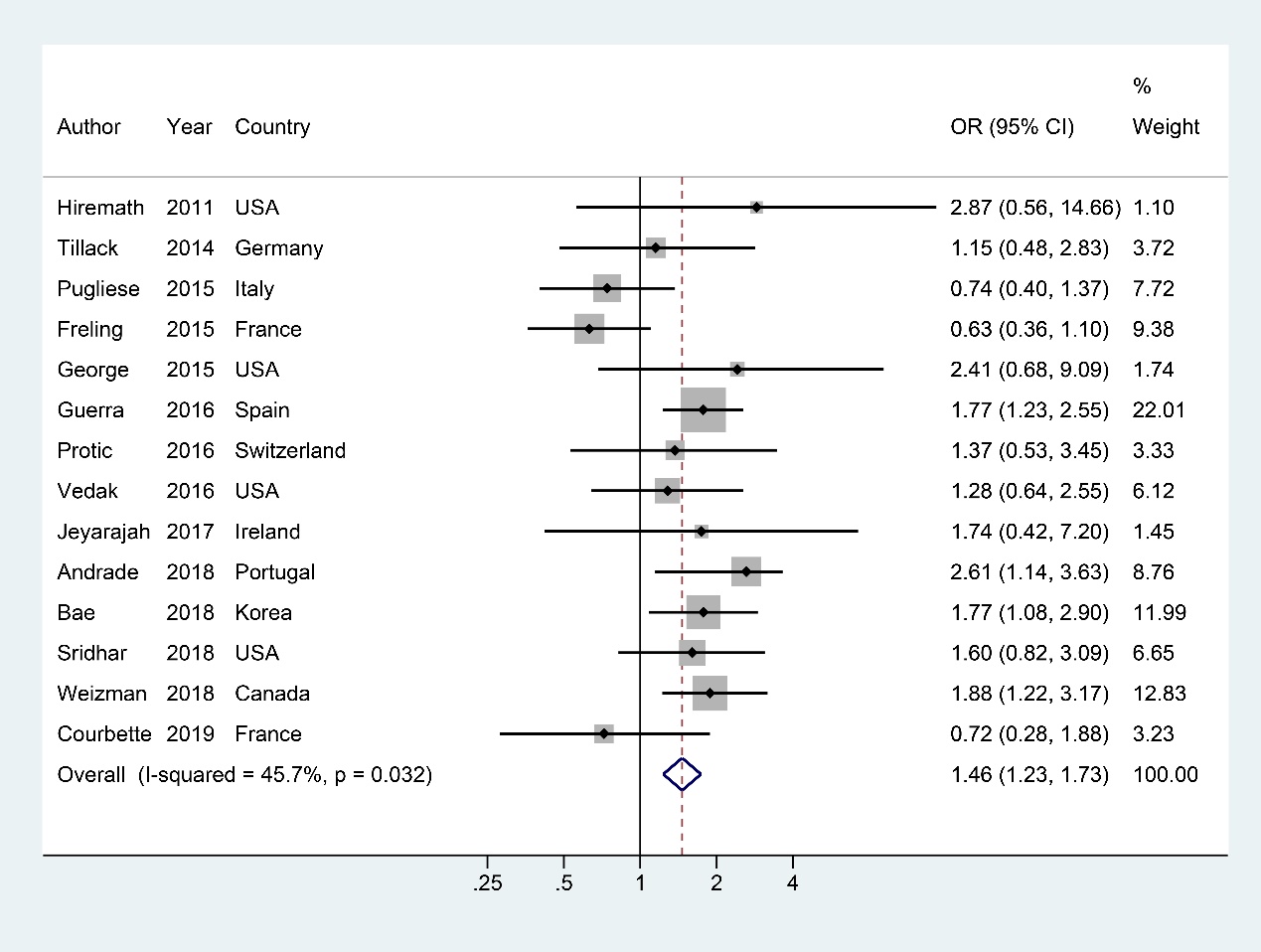
**Figure S10.** Forest plots of the odds ratio for female


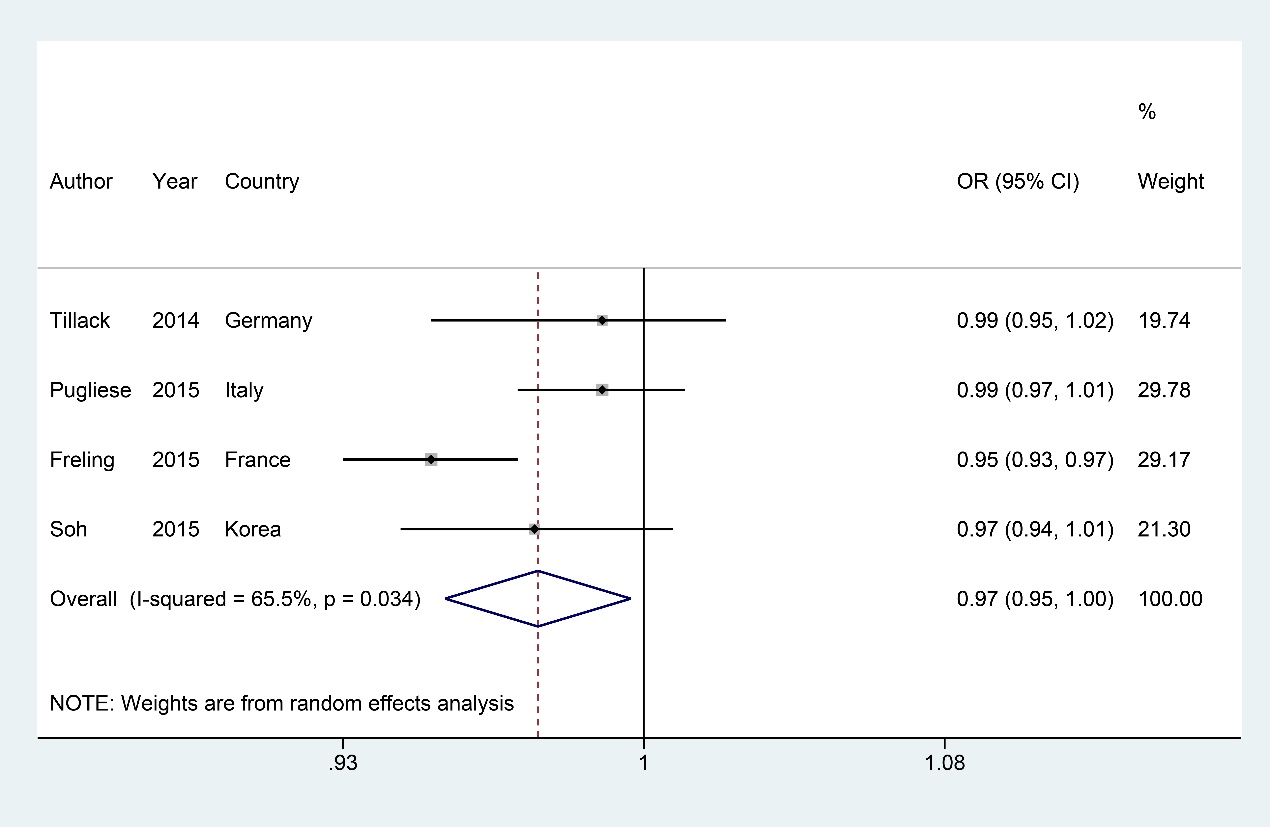
**Figure S11.** Forest plots of the odds ratio for age of anti-TNF therapy


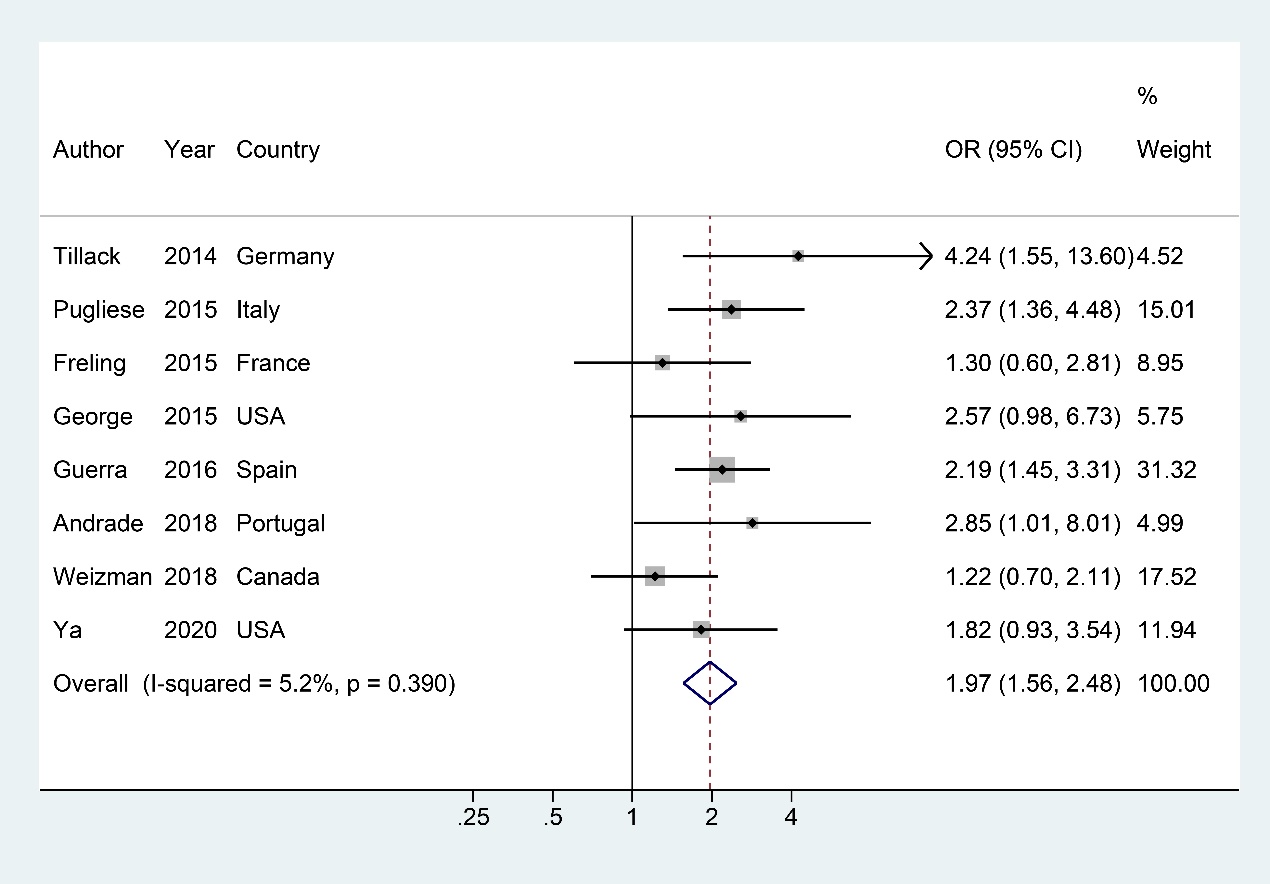
**Figure S12.** Forest plots of the odds ratio for smoking


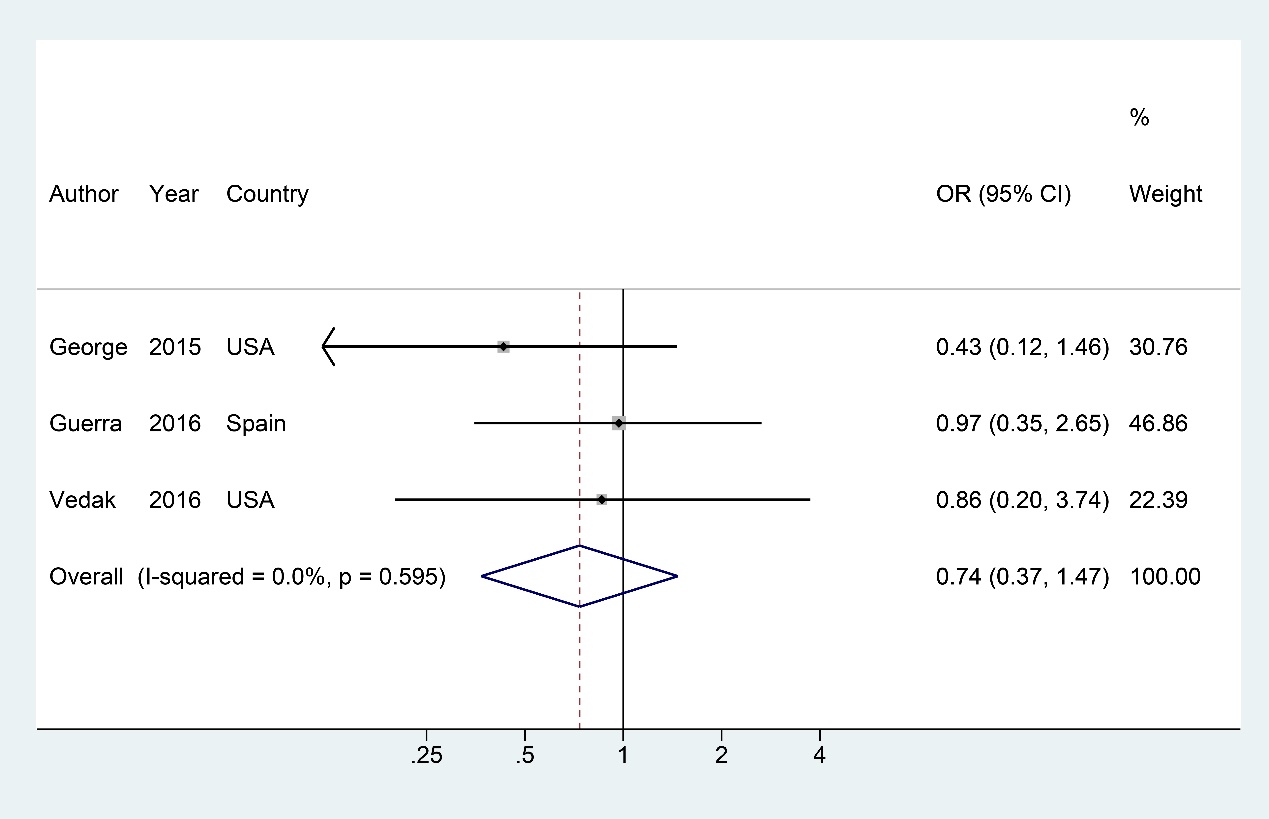
**Figure S13.** Forest plots of the odds ratio for white


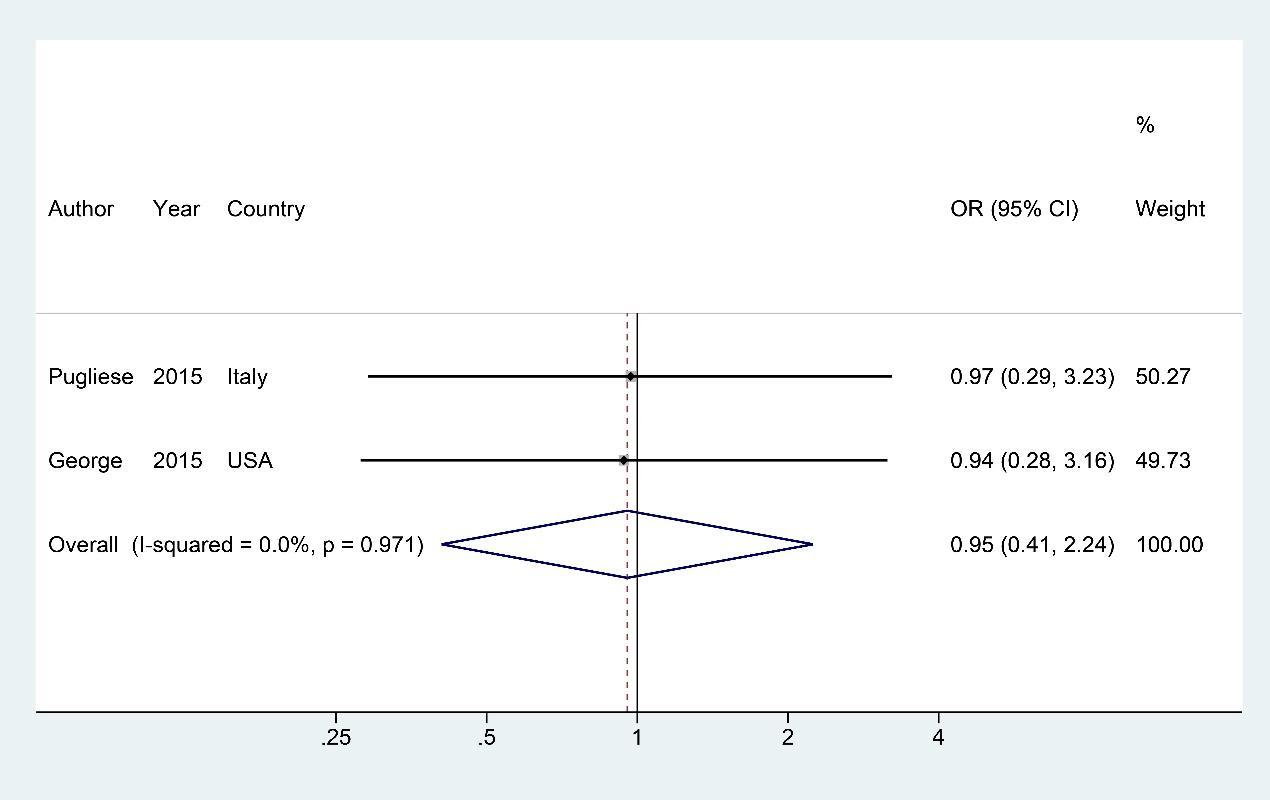
**Figure S14.** Forest plots of the odds ratio for obesity


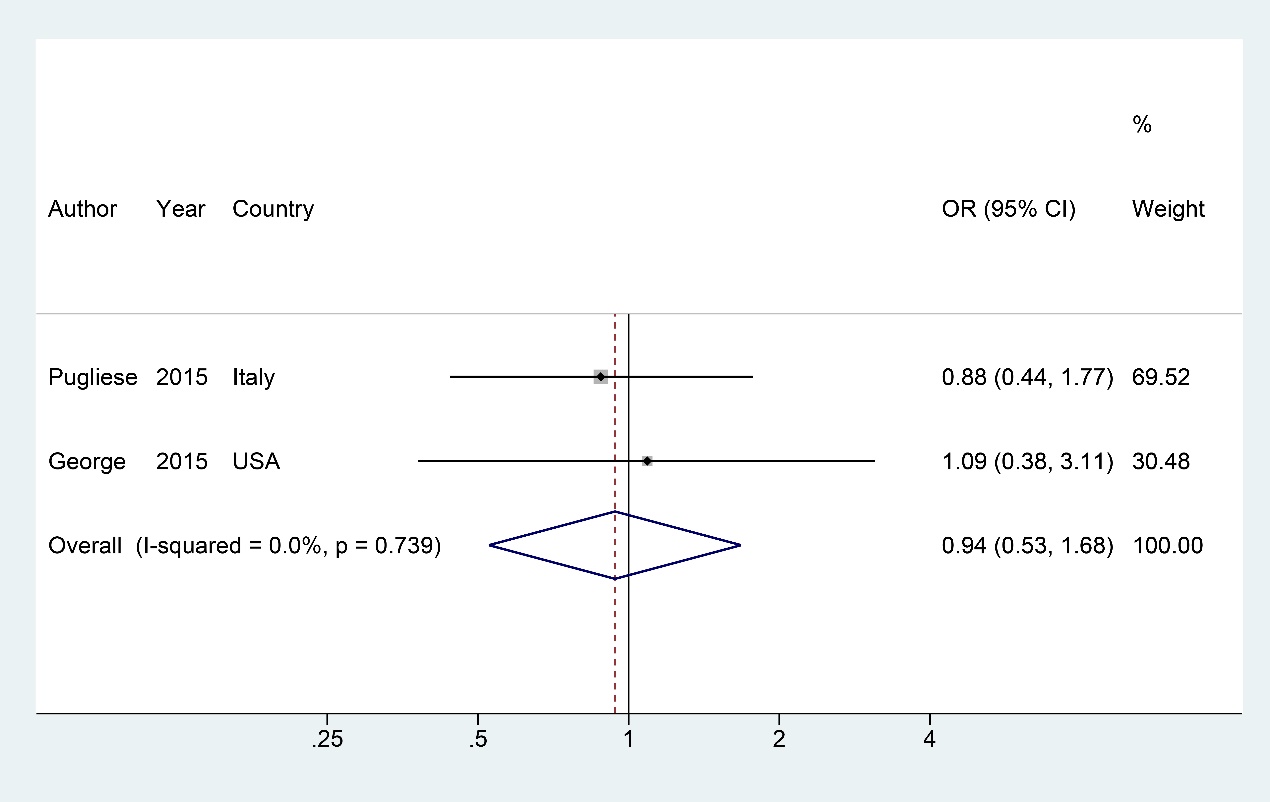
**Figure S15.** Forest plots of the odds ratio for overweight


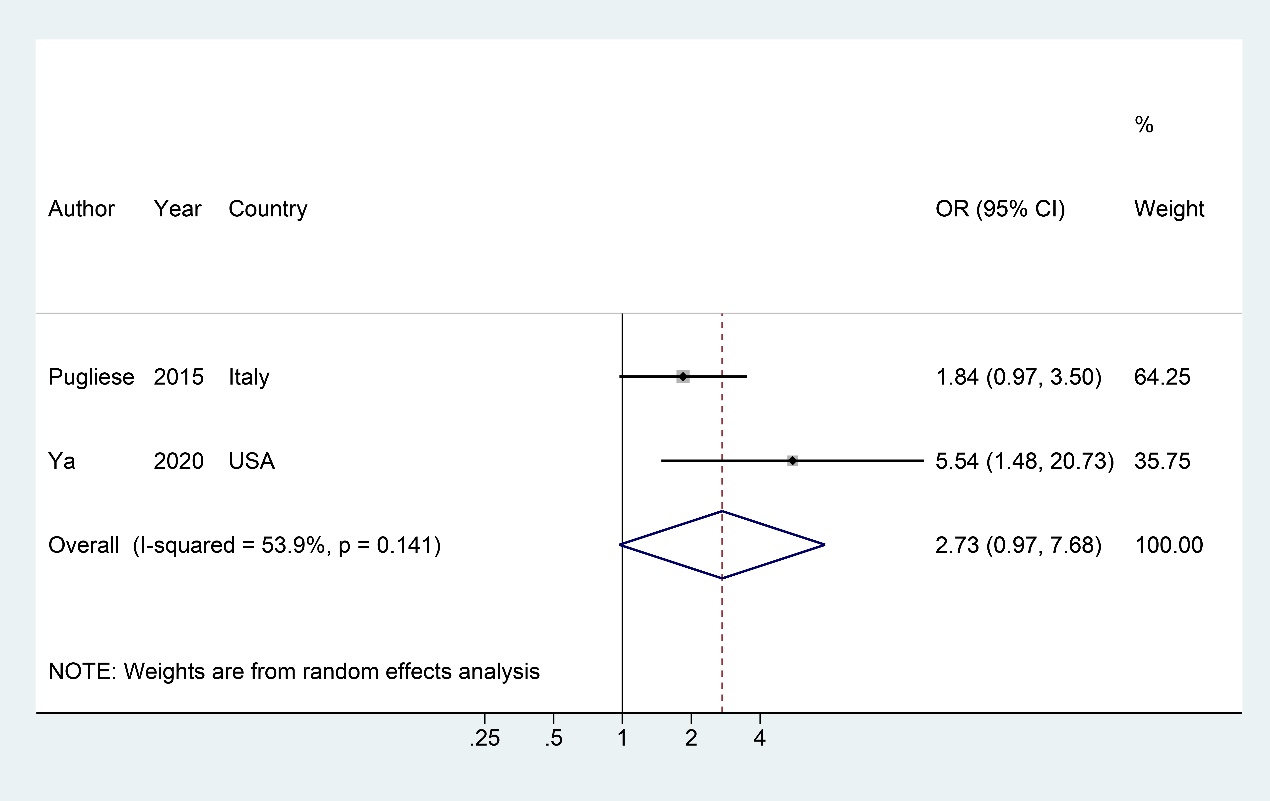
**Figure S16.** Forest plots of the odds ratio for family history of PsO


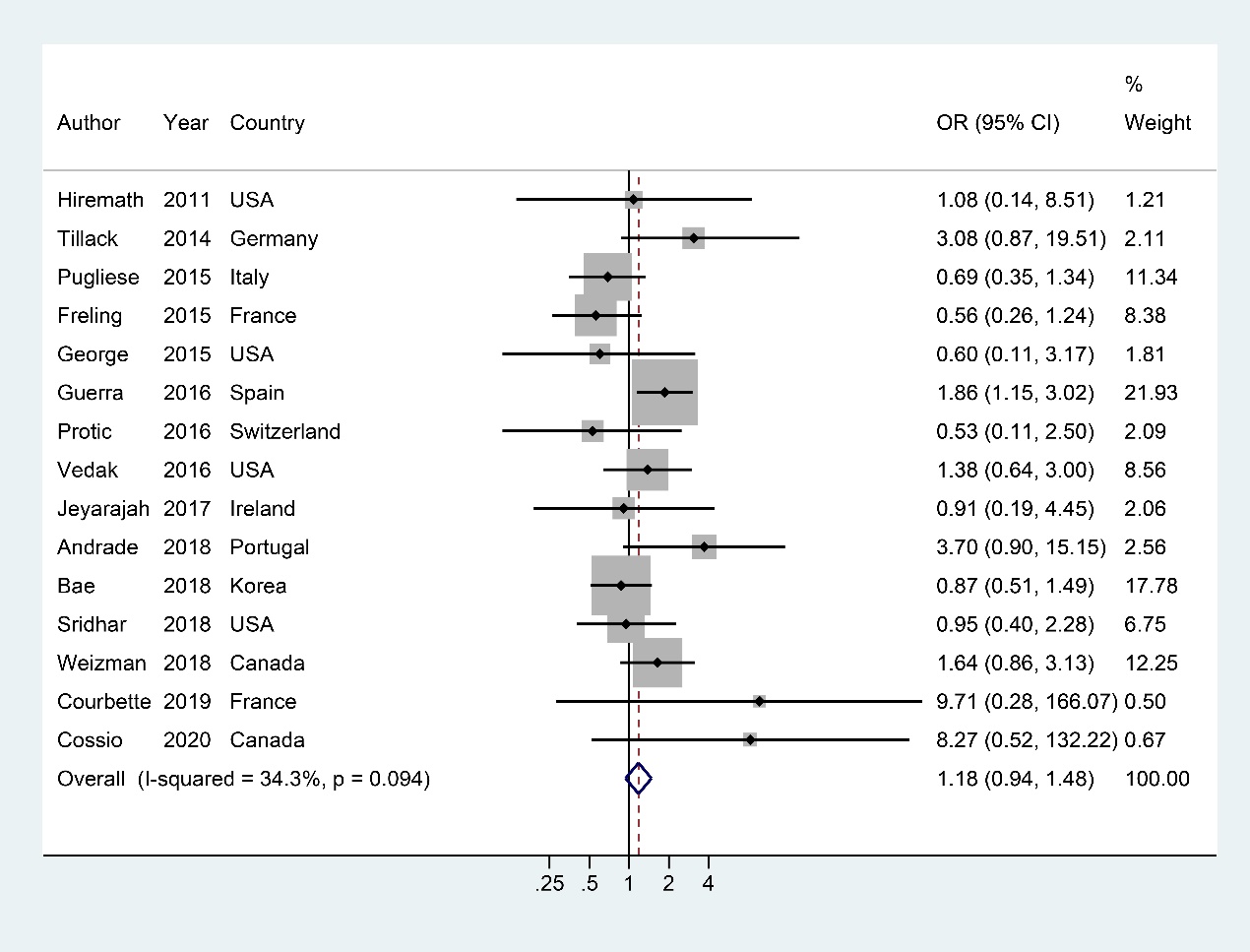
**Figure S17.** Forest plots of the odds ratio for CD (vs. UC)


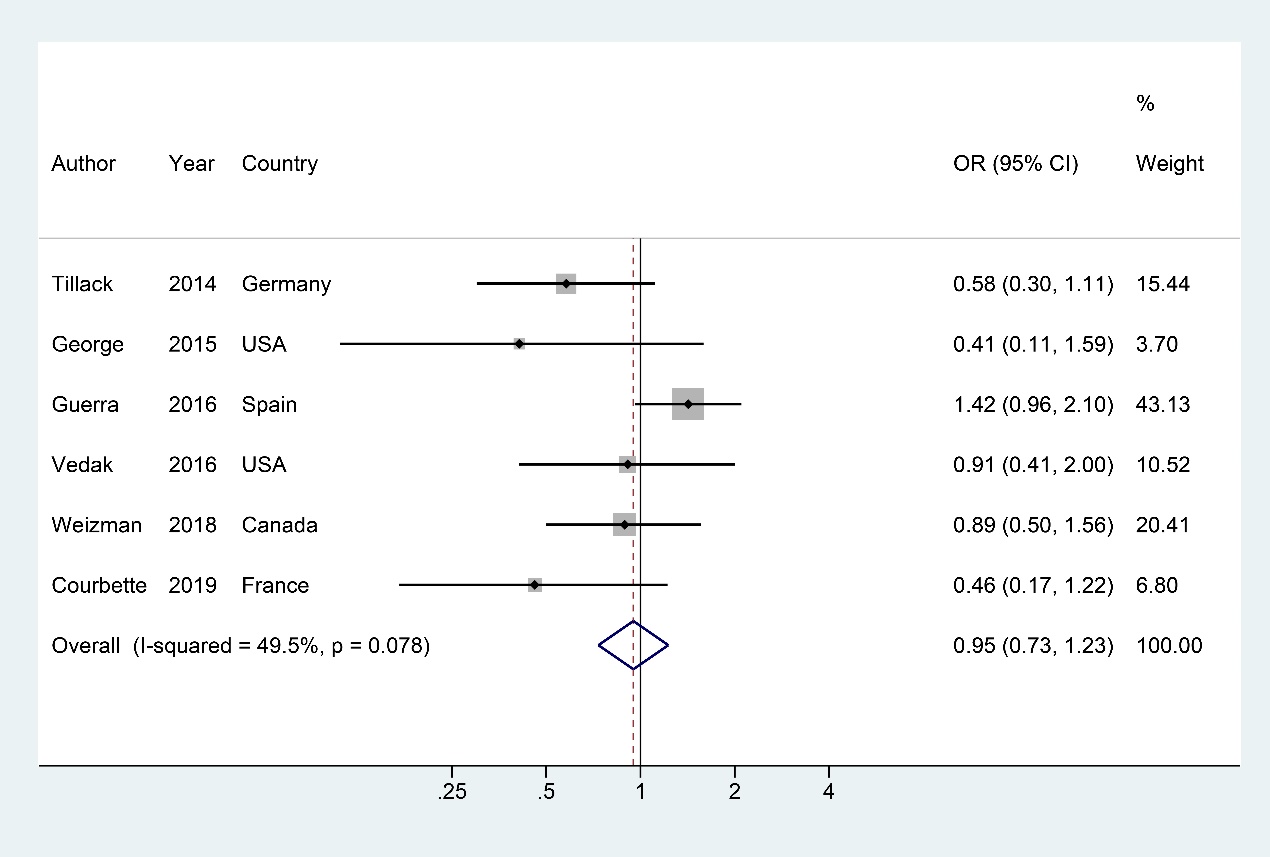
**Figure S18.** Forest plots of the odds ratio for inflammatory CD


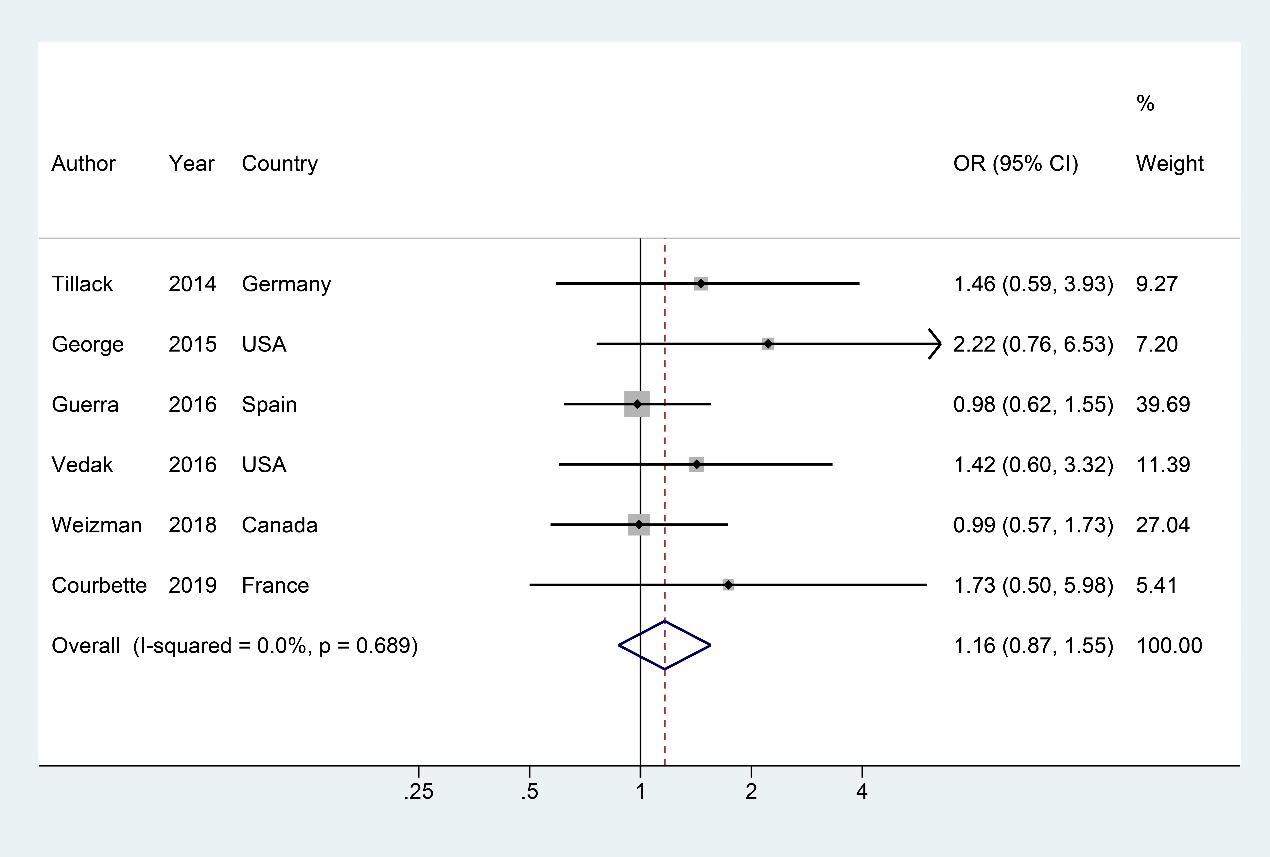
**Figure S19.** Forest plots of the odds ratio for stricturing CD


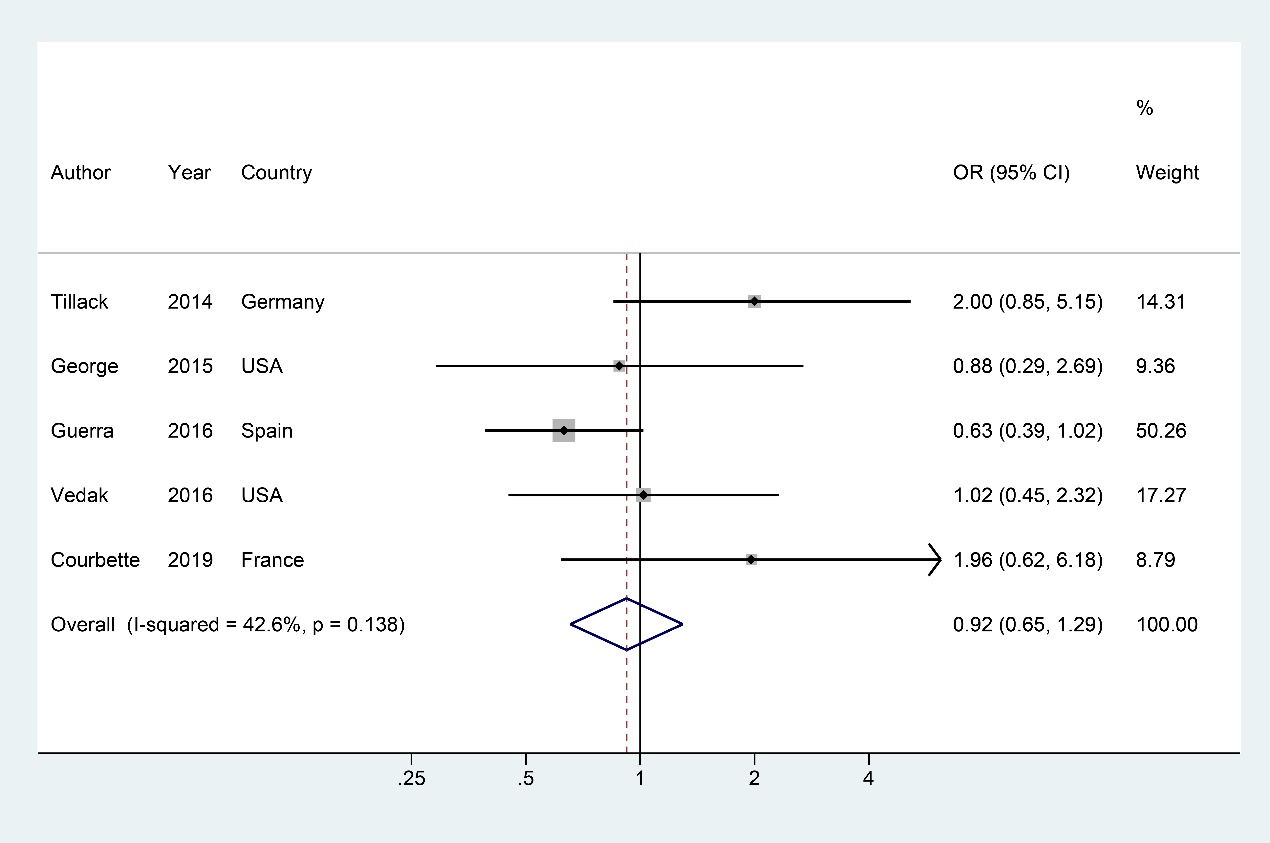
**Figure S20.** Forest plots of the odds ratio for penetrating CD


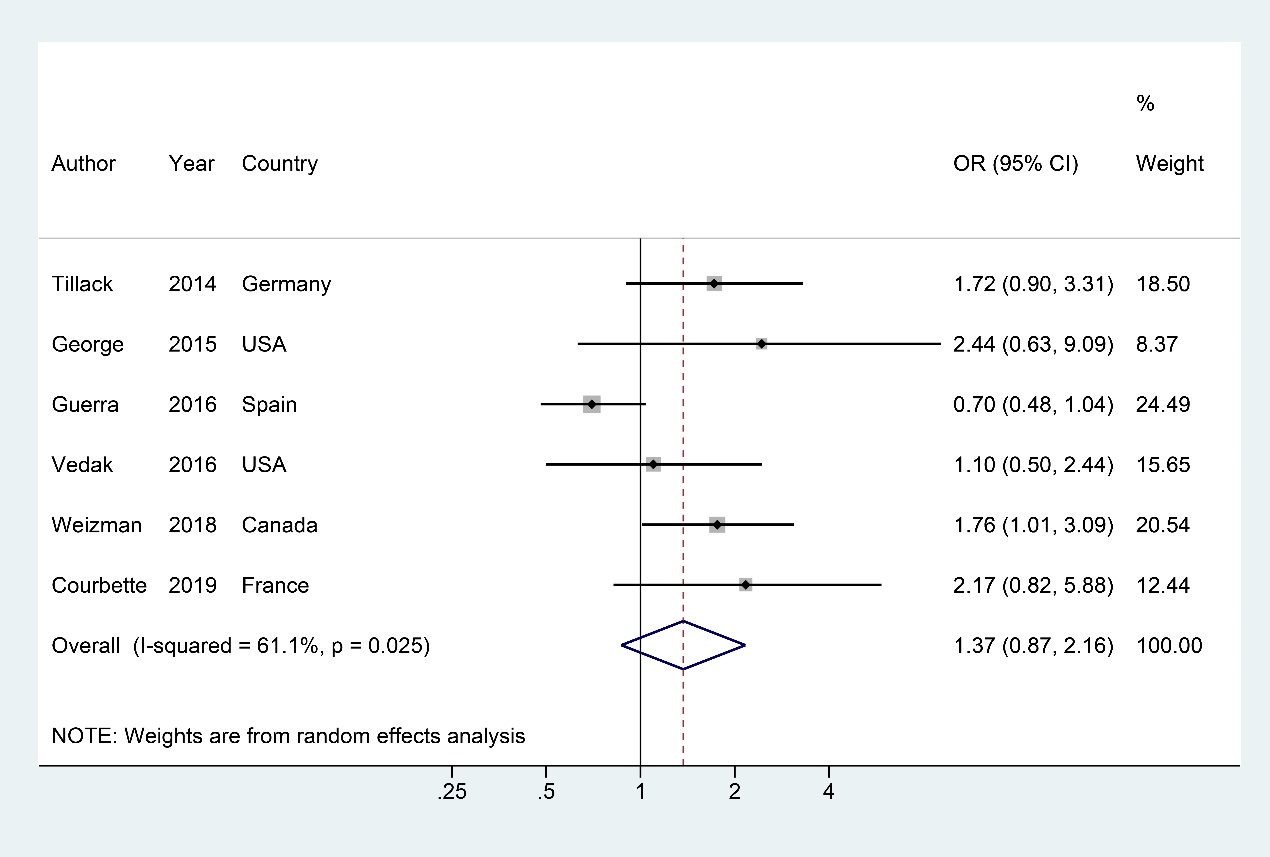
**Figure S21.** Forest plots of the odds ratio for stricturing/penetrating CD


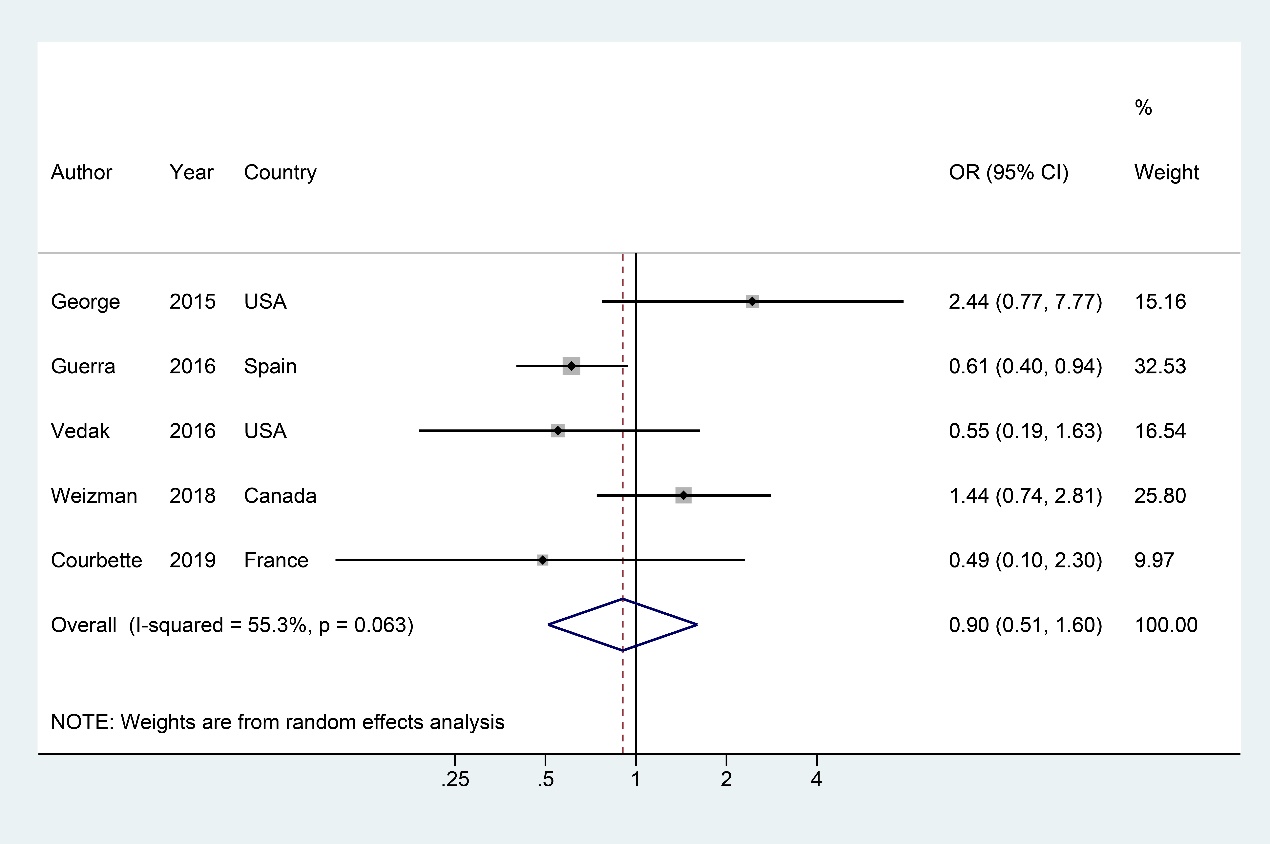
**Figure S22.** Forest plots of the odds ratio for terminal Ileum CD


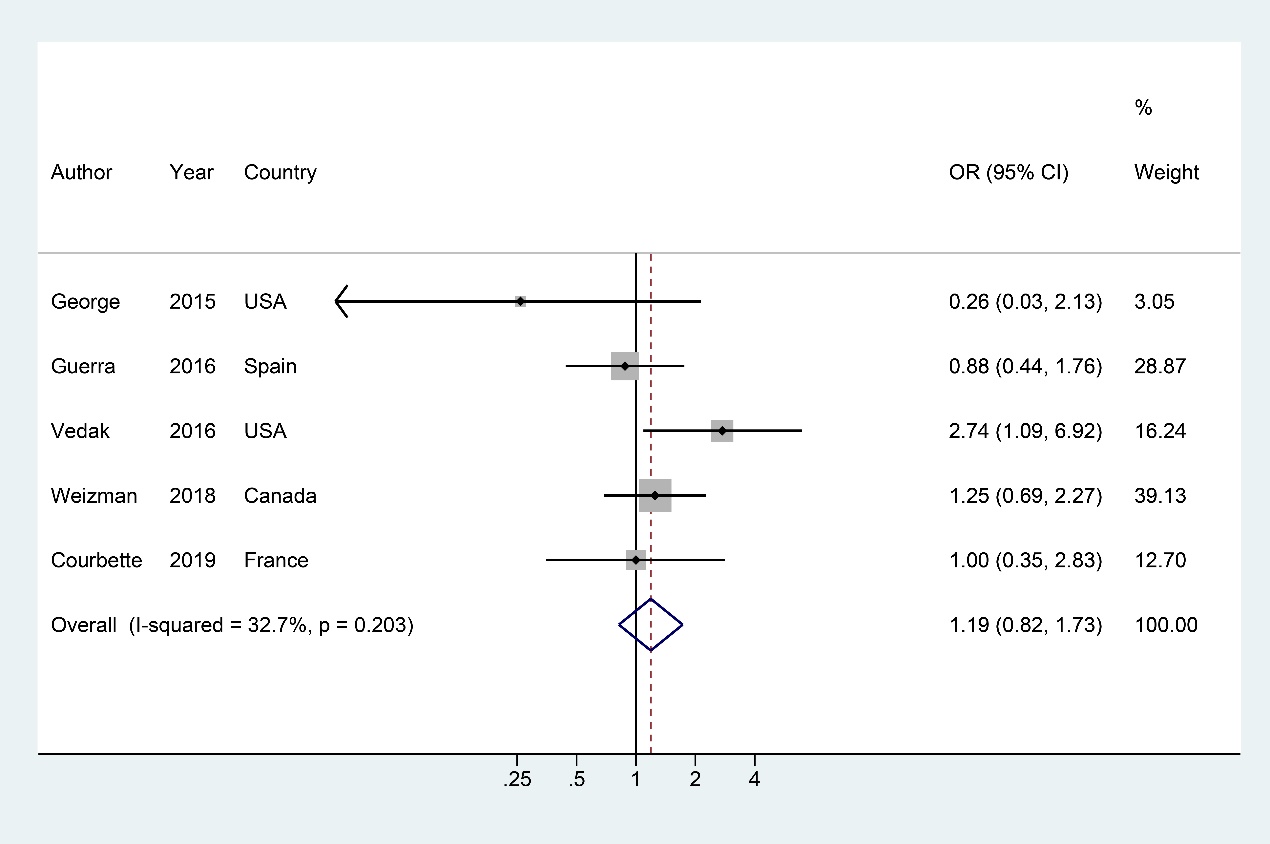
**Figure S23.** Forest plots of the odds ratio for colon CD


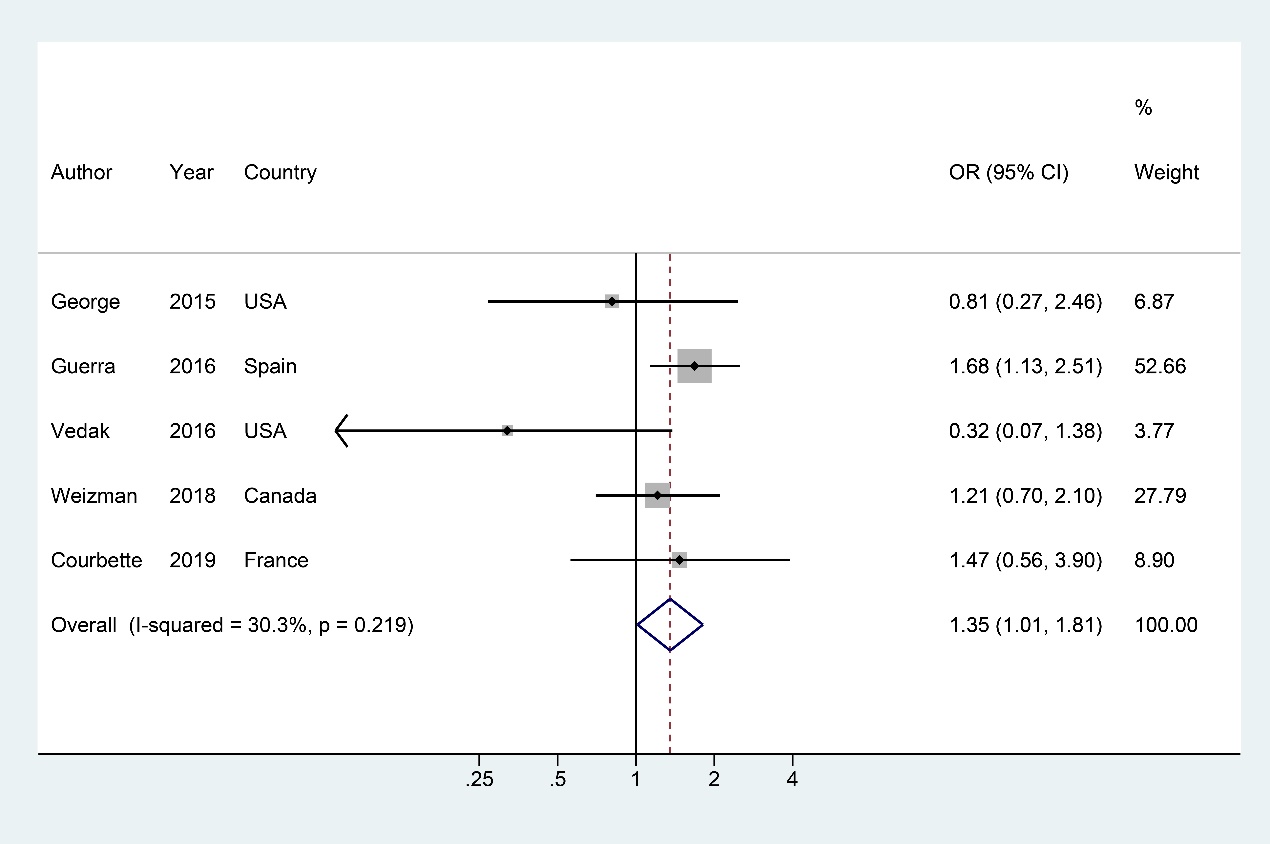
**Figure S24.** Forest plots of the odds ratio for ileocolonic CD


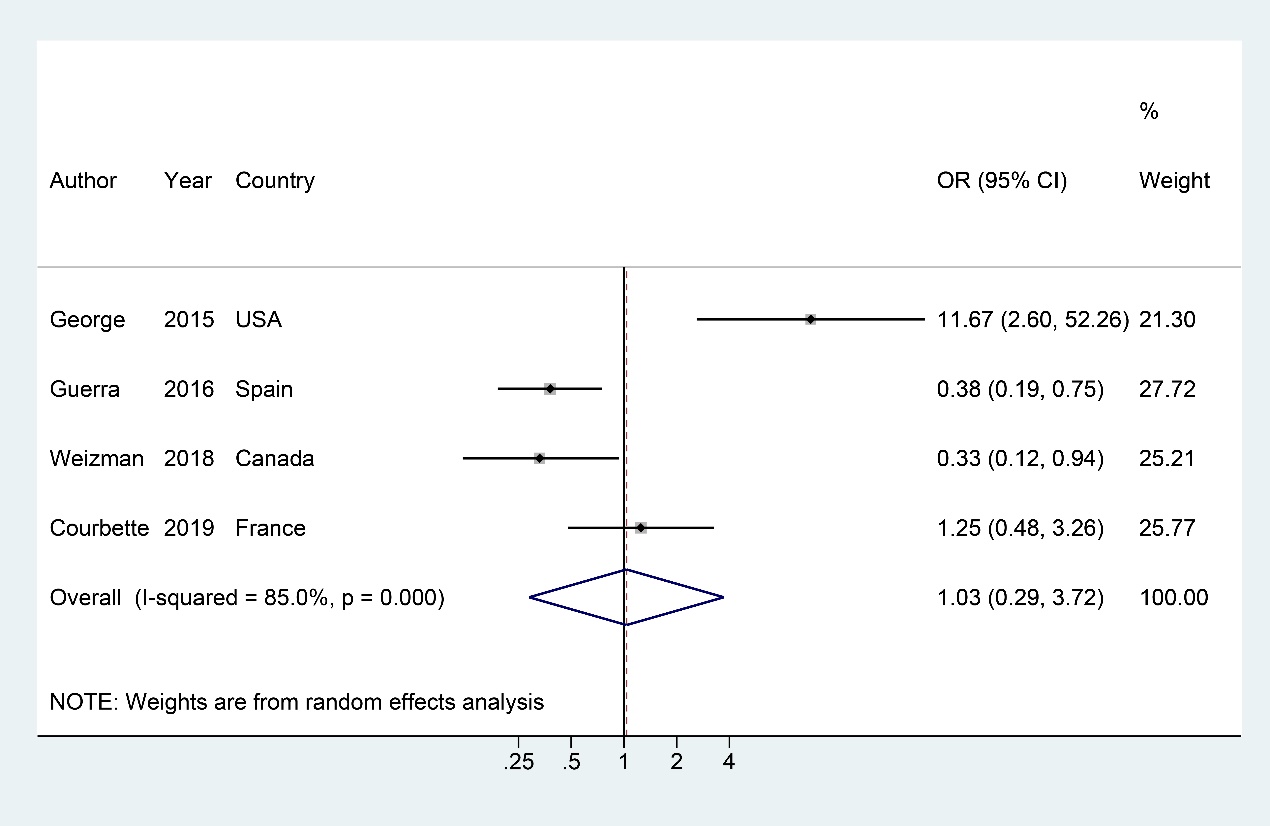
**Figure S25.** Forest plots of the odds ratio for upper tract CD


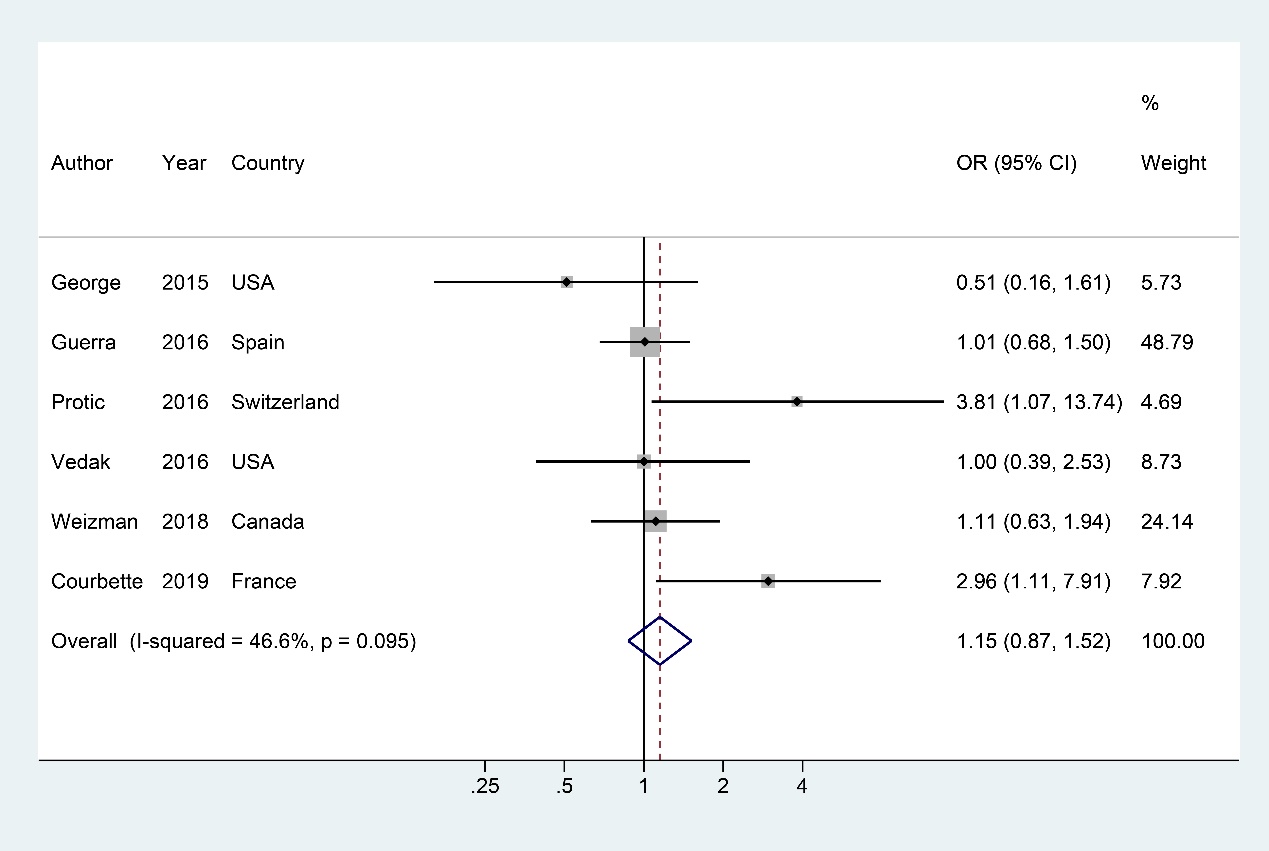
**Figure S26.** Forest plots of the odds ratio for perianal CD


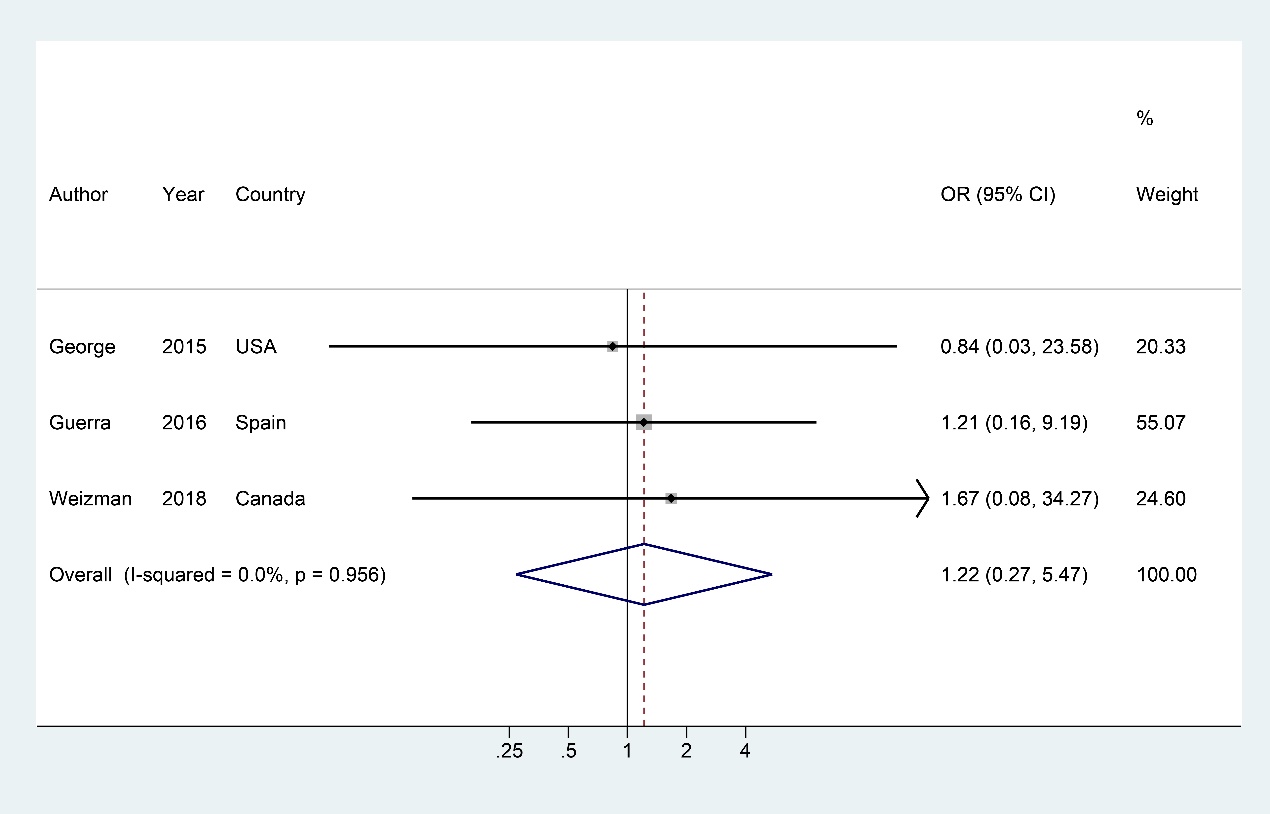
**Figure S27.** Forest plots of the odds ratio for rectum UC


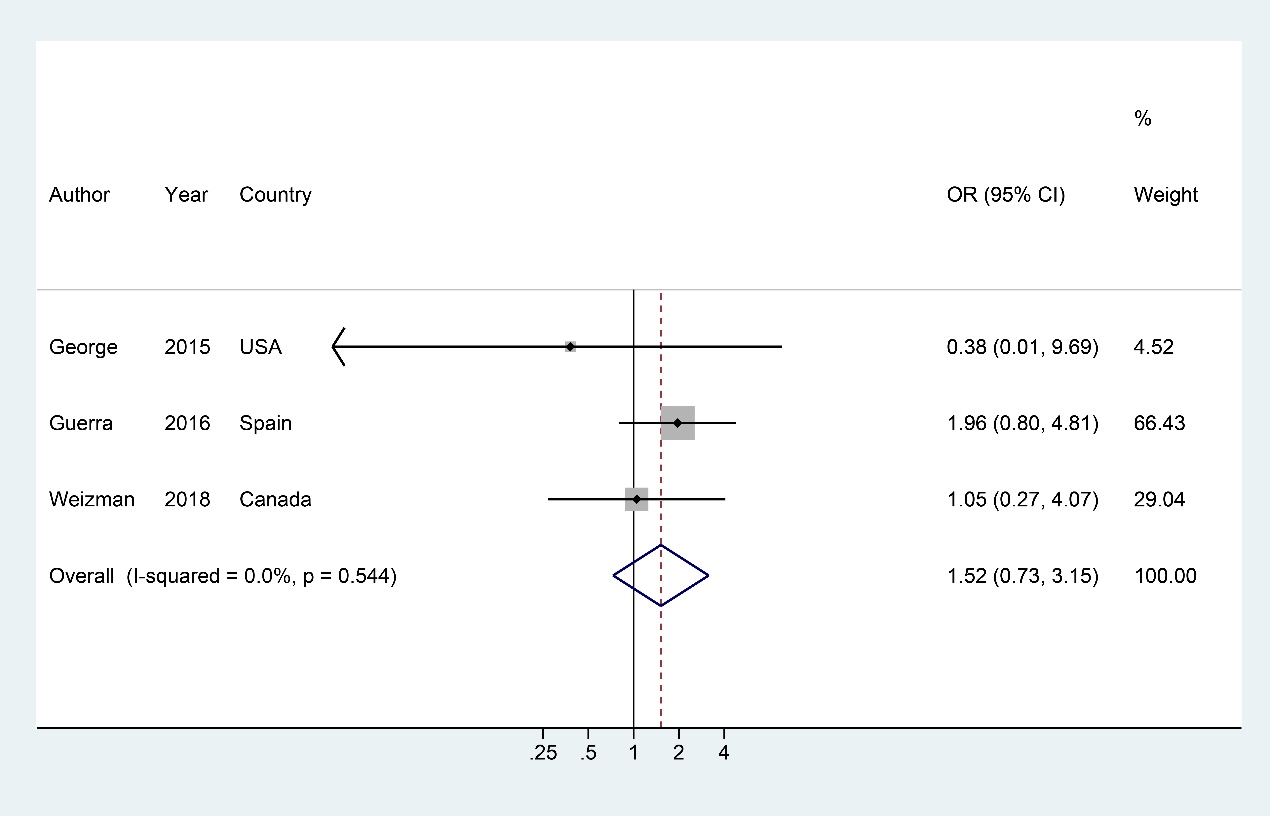
**Figure S28.** Forest plots of the odds ratio for left sided UC


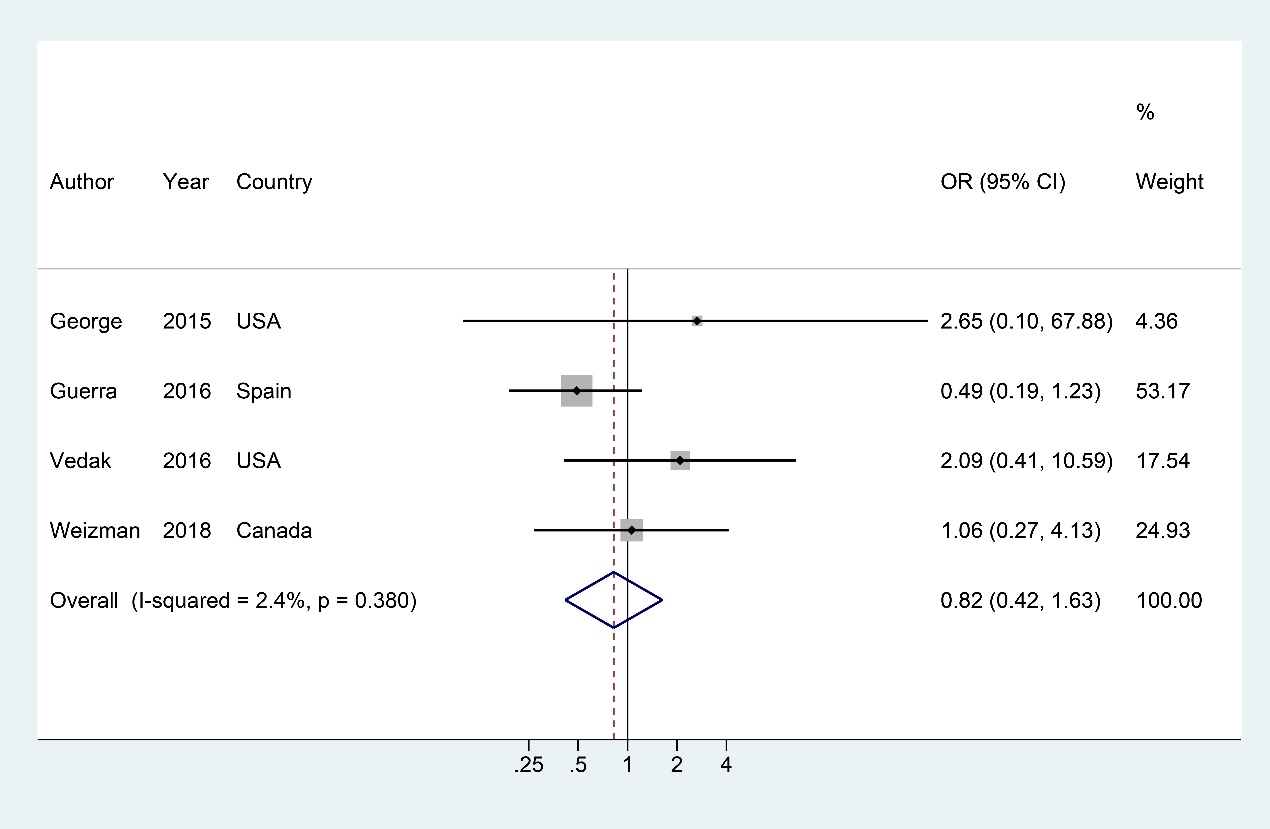
**Figure S29.** Forest plots of the odds ratio for extensive UC


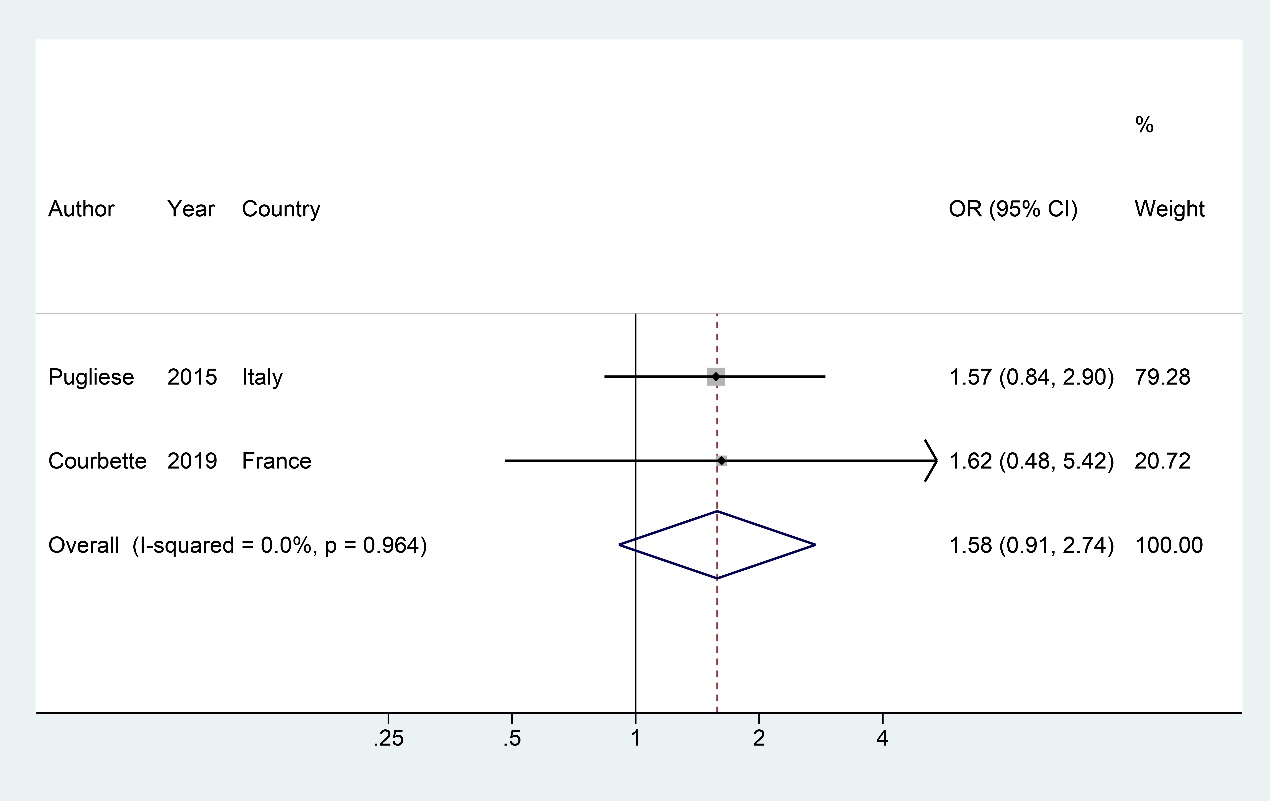
**Figure S30.** Forest plots of the odds ratio for extra-intestinal manifestations


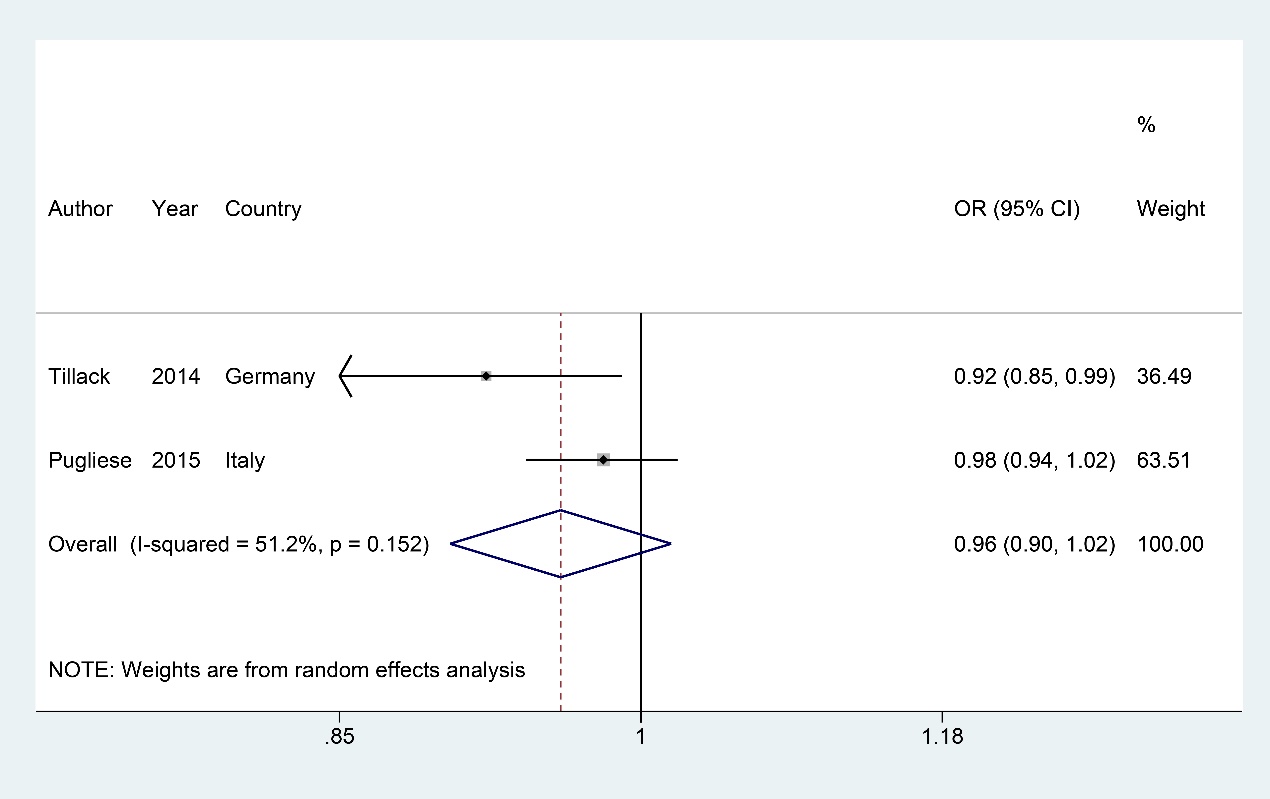
**Figure S31.** Forest plots of the odds ratio for IBD duration


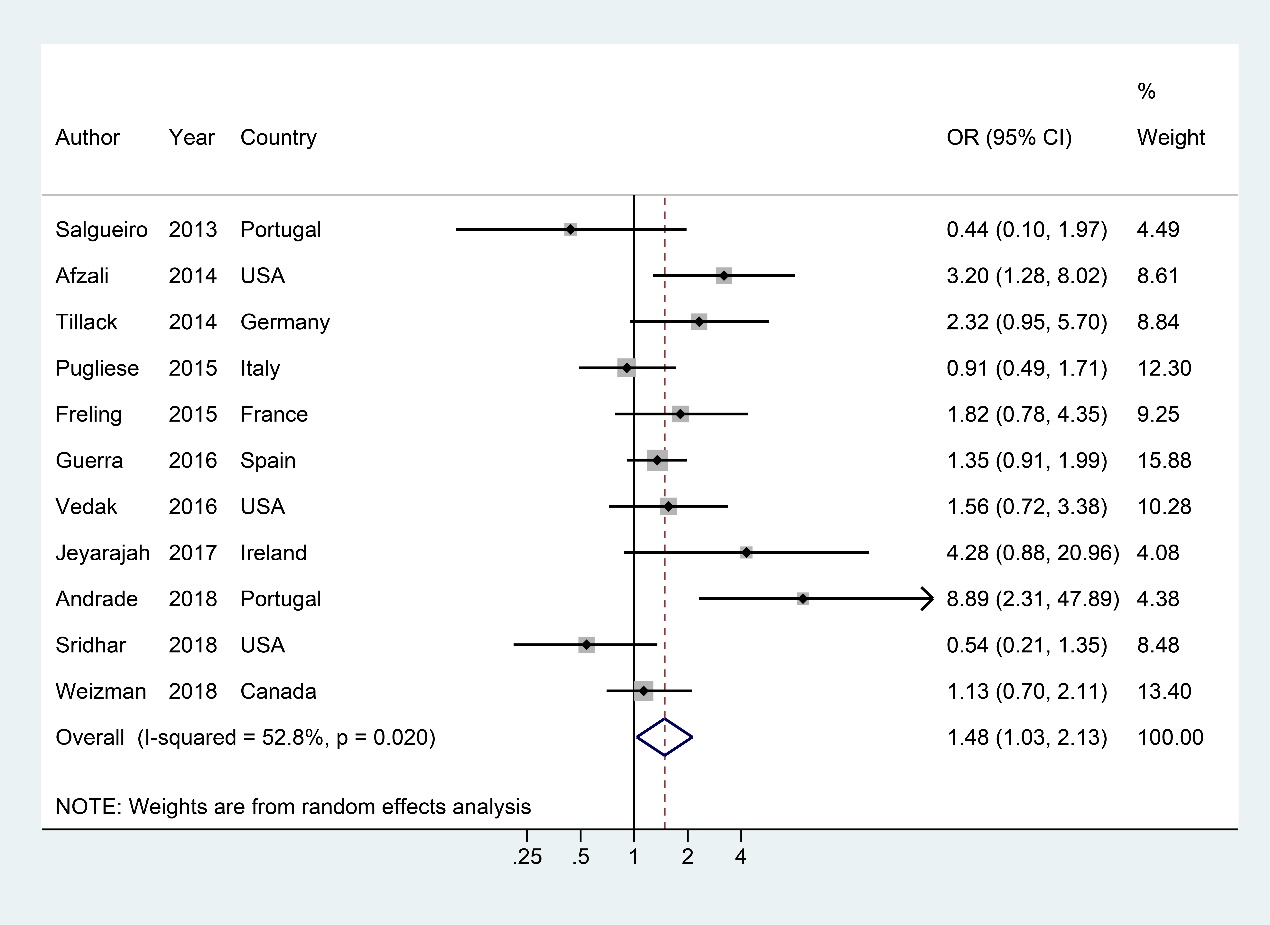
**Figure S32.** Forest plots of the odds ratio for adalimumab (vs. infliximab).


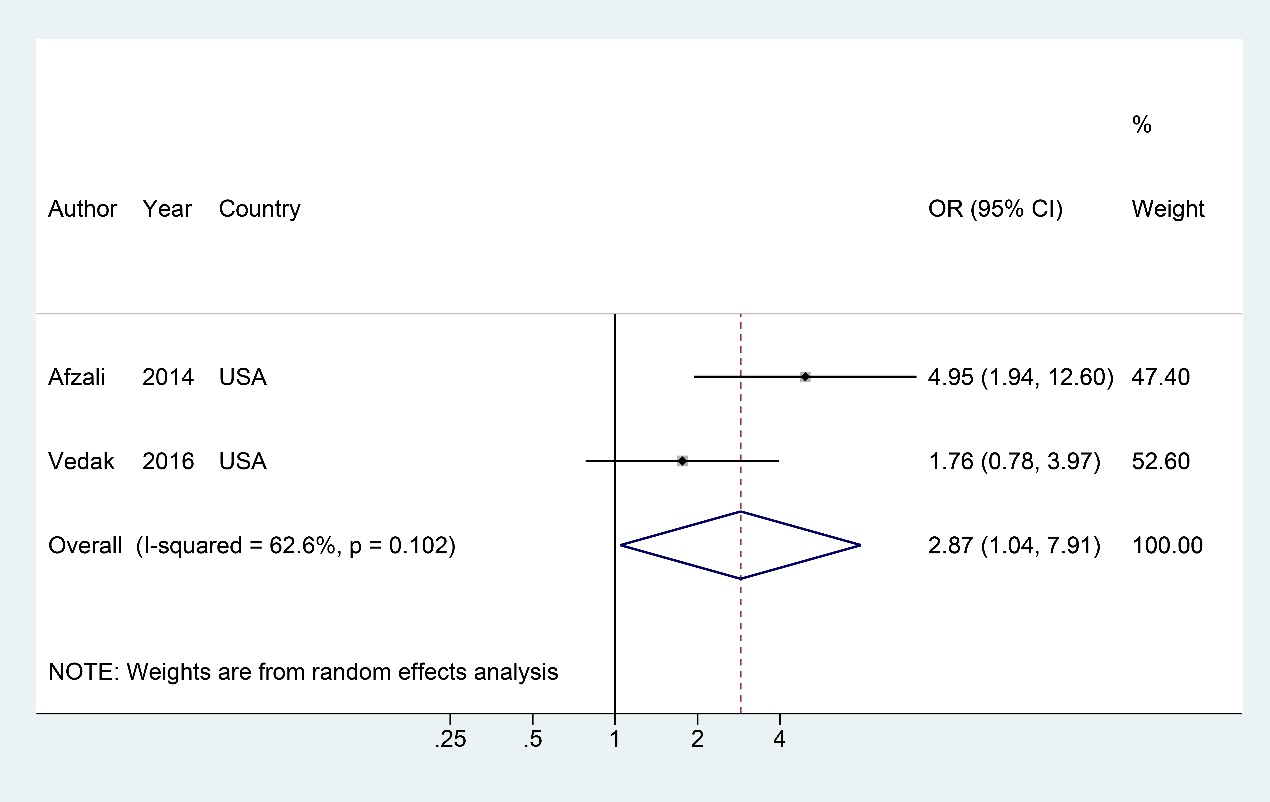
**Figure S33.** Forest plots of the odds ratio for certolizumab (vs. infliximab)


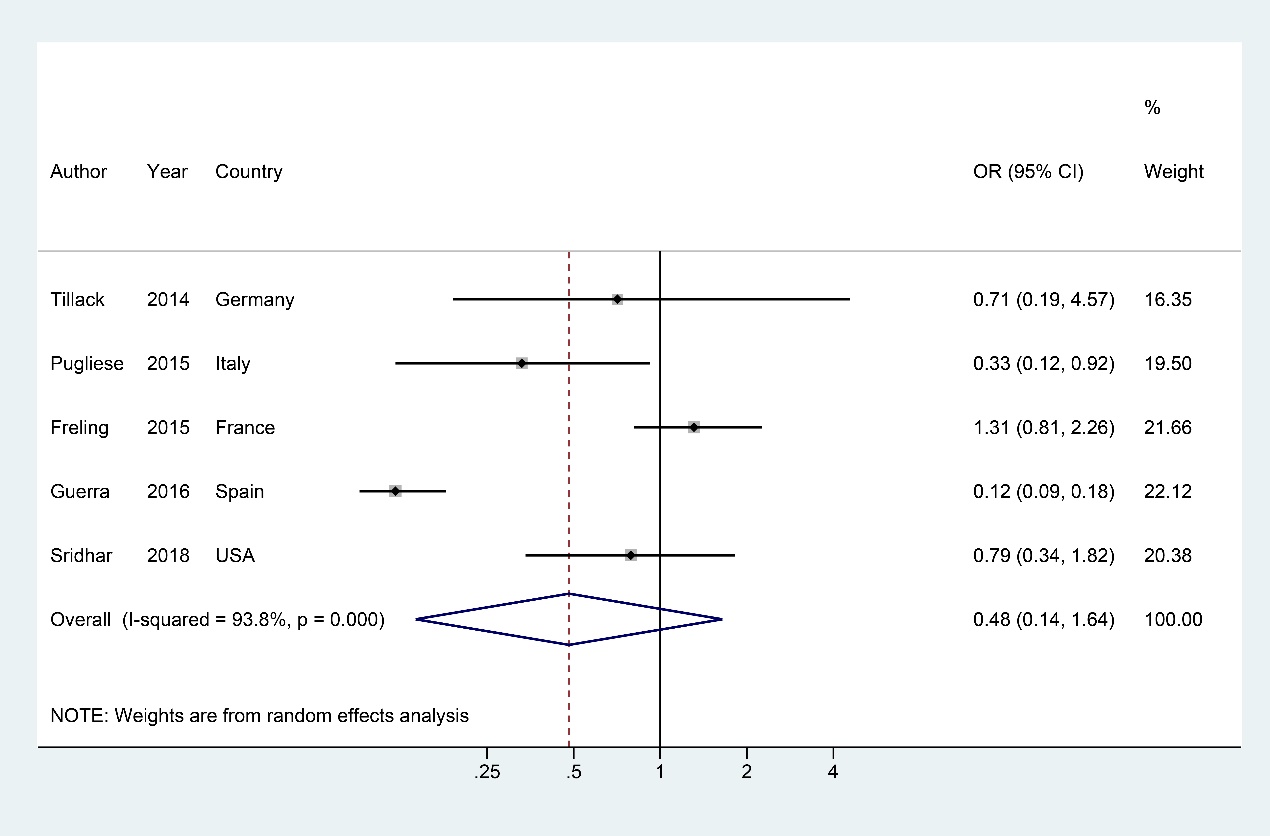
**Figure S34.** Forest plots of the odds ratio for concomitant immunosuppressants
